# Intron Editing Reveals *SNORD*-Dependent Maturation of the Small Nucleolar RNA Host Gene *GAS5* in Human Cells

**DOI:** 10.3390/ijms242417621

**Published:** 2023-12-18

**Authors:** Anastasiya Matveeva, Dmitry Vinogradov, Evgenii Zhuravlev, Dmitriy Semenov, Valentin Vlassov, Grigory Stepanov

**Affiliations:** Institute of Chemical Biology and Fundamental Medicine, Siberian Branch of the Russian Academy of Sciences, Novosibirsk 630090, Russia; anastasiya.maatveeva@gmail.com (A.M.); dm1vin52@gmail.com (D.V.); evgenijur@gmail.com (E.Z.); semenov@niboch.nsc.ru (D.S.);

**Keywords:** snoRNA, lncRNA, GAS5, box C/D snoRNA, RNA modification, genome editing, CRISPR/Cas9, alternative splicing, epitranscriptomics

## Abstract

The *GAS5* gene encodes a long non-coding RNA (lncRNA) and intron-located small nucleolar RNAs (snoRNAs). Its structure, splice variants, and diverse functions in mammalian cells have been thoroughly investigated. However, there are still no data on a successful knockout of *GAS5* in human cells, with most of the loss-of-function experiments utilizing standard techniques to produce knockdowns. By using CRISPR/Cas9 to introduce double-strand breaks in the terminal intronic box C/D snoRNA genes (*SNORD*s), we created monoclonal cell lines carrying continuous deletions in one of the *GAS5* alleles. The levels of *GAS5*-encoded box C/D snoRNAs and lncRNA GAS5 were assessed, and the formation of the novel splice variants was analyzed. To comprehensively evaluate the influence of specific *SNORD* mutations, human cell lines with individual mutations in *SNORD74* and *SNORD81* were obtained. Specific mutations in *SNORD74* led to the downregulation of all *GAS5*-encoded SNORDs and GAS5 lncRNA. Further analysis revealed that *SNORD74* contains a specific regulatory element modulating the maturation of the *GAS5* precursor transcript. The results demonstrate that the maturation of GAS5 occurs through the m6A-associated pathway in a *SNORD*-dependent manner, which is a quite intriguing epitranscriptomic mechanism.

## 1. Introduction

**Figure 1:**
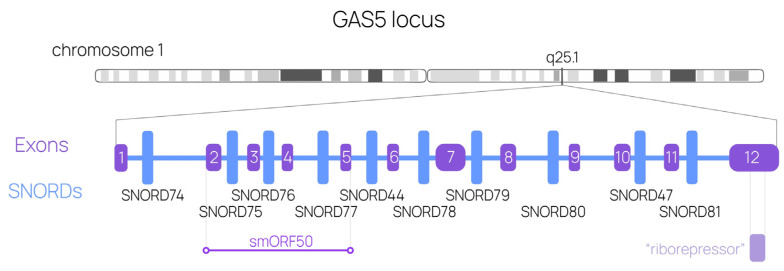
Human *GAS5* locus structure. The chromosome localization is presented, as is the exon-intron structure with 10 intronic SNORDs and the riborepressor mimicking the GR response element.

The *GAS5* (Growth Arrest-Specific 5) gene has been discovered to be among the six specific genes expressed in G0-cells (cells in the pre-replicative phase) [1]. While the five other genes are transcribed into mRNAs, *GAS5* produces a long non-coding RNA. The human *GAS5* transcription unit is 4 kb long and quite complex due to the large numbers of exons, alternative promoters, and alternative splicing events. The *GAS5* locus itself exhibits a peculiar structure, comprising 12 exons that can generate up to 24 varying mature lncRNA isoforms through alternative splicing. It also includes 10 intron-encoded box C/D small nucleolar RNAs (box C/D snoRNAs), a 3′-exon-encoded riborepressor, and several conserved small open reading frames (smORFs; Figure 1) [2,3,4]. Such a diverse structure allows for a wide range of functions. GAS5 lncRNA expression has been shown to be decreased in various cancer types, suggesting its putative tumor-suppressing role [2,5,6,7]. Although smORFs are detected in the *GAS5* locus, there are still no data on the corresponding peptides. In addition, the only known interaction of GAS5 transcripts with protein partners occurs through the 3′-located riborepressor. Its stem structure resembles DNA targets for glucocorticoid receptor (GR), thus enabling the interaction between GAS5 and GR [8,9]. GAS5 lncRNA is capable of interacting with multiple miRNAs as well as regulating their levels [10,11,12]. Among other suggested functions are the regulation of gene expression through promoter binding and exosomal cell-to-cell communication [13,14,15]. The question of whether GAS5 lncRNA regulates multiple genes, thereby contributing to its tumor-suppressive phenotype, remains unanswered, leaving a wide area for investigation [3].

Although many studies have applied various techniques, such as the use of small interfering RNAs and short-hairpin RNAs, to create GAS5 lncRNA knockdowns, there are no data on the knockout of this gene in human cells [16,17,18,19,20,21]. The lack of data might indicate that standard genome-editing approaches fail to delete the whole gene, from the first to the last exon. An interesting alternative approach could involve editing the *GAS5* introns specifically at the points where small nucleolar RNA genes localize.

**Figure 2:**
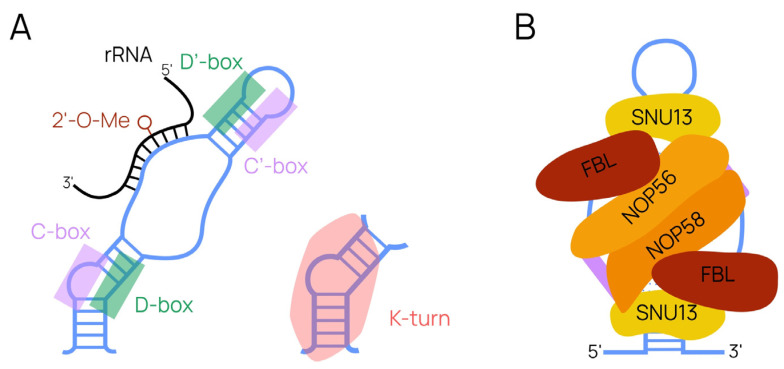
Box C/D-snoRNA structure. (**A**) Key elements crucial for snoRNA maturation and functioning are shown in the form of conserved boxes and the K-turn structure formed by them. (**B**) A formed ribonucleoprotein with canonical protein partners [26].

Small nucleolar RNAs represent a class of small non-coding RNAs that participate in the post-transcriptional modification of ribosomal RNA (rRNA) in eukaryotes. Two subclasses exist, box C/D and box H/ACA snoRNAs, and these subclasses provide 2′-O-methylation (2′-O-Me) and pseudouridylation of rRNA, respectively [22]. Both of these modifications are abundant throughout eukaryotic rRNA, with a specific snoRNA “assigned” to modify a particular nucleotide [23]. Box C/D snoRNAs (also referred to as SNORDs) can facilitate up to two modifications, as their structure includes one or two 10–21-nucleotide guide sequences. These structural elements are complementary to the specific region of rRNA where the modification occurs. Essential structural C (RUGAUGA, where R = purine) and D (CUGA) boxes participate in the formation of functionally active ribonucleoprotein complexes. The stem-bulge-stem “kink-turn” structure (K-turn) is formed by boxes C and D and terminal regions of snoRNA (Figure 2A) [24]. Further, core snoRNA proteins (Snu13, NOP56, NOP58, and fibrillarin) recognize the K-turn and initiate the formation, processing, and localization of a mature small nucleolar ribonucleoprotein (snoRNP), all of which is required for its proper functioning in cells (Figure 2B) [25].

While snoRNA’s role as a “guide” for rRNA 2′-O-methylation and pseudouridylation has long been recognized, many novel functions are still being discovered [27]. Among those are rRNA acetylation [28], regulation of alternative splicing [28,29,30,31], complex autoregulatory events [32], regulation of intracellular cholesterol trafficking, exome recruitment and chromatin remodeling, modulation of metabolic and oxidative stress, and memory consolidation and learning [25,33,34,35,36,37,38,39,40]. In eukaryotes, snoRNAs that lack a guide sequence or have no characteristic complementary rRNA region have been termed ‘orphan’ snoRNAs [41]. Such orphan snoRNAs can participate in tRNA methylation [41], modulation of the 3′-processing of mRNA [42,43], post-transcriptional mRNA silencing [44,45], and the development of disorders [46,47]. It is now well known that some snoRNAs can be processed into stable short RNA forms named snoRNA-derived snoRNAs (sdRNAs) [48]. Several sdRNAs possess microRNA-like gene-silencing activity [49,50] and regulate alternative splicing [51,52]. So-called sno-lncRNAs—long RNA species that contain snoRNA—have been observed as well [53].

Among many varying RNA modifications in eukaryotes, N6-methyladenosine (m6A) is the most abundant in mRNA [54]. It is added by the methyltransferase complex consisting of m6A-methyltransferases METTL3, METTL14, METTL16 (“writers”) and their partners, WTAP, VIRMA, ZC3H13, and RBM15. The modification can be removed by m6A demethylases (“erasers”, FTO and ALKBH5) and recognized by m6A-binding proteins (“readers”, YTHDF1/2/3, YTHDC1/2, IGF2BPs, hnRNPA2B1, hnRNPC, hnRNPG, etc.). Various stages of RNA metabolism are m6A-dependent, indicating its role as an important fine-tuner of cellular processes [54,55,56,57,58,59]. Studies on m6A involvement in various molecular mechanisms revealed interesting crosstalk between m6A modification and alternative splicing [60,61,62]. Splicing can be modulated through either recruiting specific RNA-binding proteins (RBPs) or disrupting the interaction of the modified pre-mRNA with its corresponding RBP. On the other hand, studies have shown that alternative splicing events can regulate the addition or recognition of m6A modifications [62]. Further research on the mechanisms behind this interplay can contribute to both fundamental knowledge and the development of therapeutic strategies.

*GAS5* introns encode ten box C/D snoRNAs, allowing for the manipulation of their genes without frameshift or disruption to the host-gene structure (Figure 1). Several studies have demonstrated the feasibility of employing genome-editing tools, particularly CRISPR/Cas9, to deplete a specific *SNORD* gene [63,64,65,66]. The effect of CRISPR-induced point mutations in *GAS5*-encoded *SNORD*s on the maturation of host-gene lncRNA has been described previously [67]. Given that it is possible to obtain a viable human cell line carrying a single depleted or modified *SNORD* gene, we targeted CRISPR/Cas9 at two terminal *SNORD* genes (*SNORD74* and *SNORD81*), aiming to fully deplete cells of 10 GAS5-encoded SNORDs and most of the *GAS5* gene itself.

In this study, we obtained viable 293FT-derived human cell lines lacking the greater part of a *GAS5* allele. The suggested strategy enabled complete suppression of GAS5 lncRNA and all intron-encoded box C/D-snoRNAs, which has not been achieved previously. Further analysis revealed that the structure of the second *GAS5* allele (without the deletion) affected the degree of downregulation of the GAS5 transcript. To assess the influence of target *SNORD* mutations, we created human cell lines carrying point mutations in either *SNORD74* or *SNORD81*. The analysis of GAS5 and snoRNA expression suggested the presence of a regulatory structure in *SNORD74* that modulates the processing of the GAS5 precursor transcript. A meta-analysis using RNA-modification databases suggested that the observed regulation may be m6A-dependent. Overall, our data supported the hypothesis that maturation of the GAS5 lncRNA precursor is *SNORD*-dependent.

## 2. Results

### 2.1. Selection and Design of Single Guide RNAs Targeted at snoRNAs

**Figure 3:**
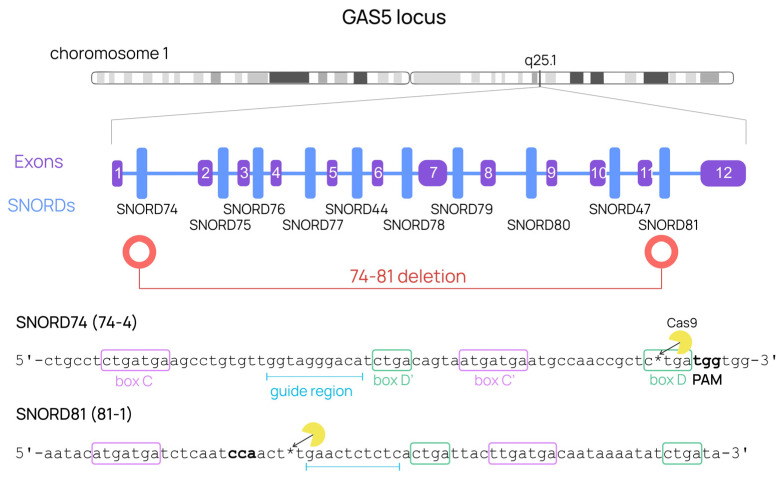
Structure of the *GAS5* gene with the targets selected for CRISPR-induced large deletions. Target *SNORD* sequences are marked with asterisks indicating the site of the DSBs expected to be generated by Cas9.

To obtain human cell lines with the depleted *GAS5* gene, CRISPR/Cas9 was targeted at the gene’s two terminal points. *SNORD74* and *SNORD81* are snoRNA genes located in *GAS5* introns 1 and 11, respectively. Although *SNORD74* contains multiple protospacer adjacent motifs (PAMs, 5′-NGG) necessary for CRISPR/Cas9-mediated editing, the PAM located close to the essential D-box was selected, as the corresponding single guide RNA (sgRNA) had previously demonstrated sufficient editing efficacy [67]. *SNORD81*, on the contrary, has not yet been tested as a genome-editing target. A single PAM located near the predicted recognition region was selected for the design of the guide RNA protospacer (Figure 3). Two sgRNAs were constructed to target the cleavage of either *SNORD74* or *SNORD81*. Each sgRNA can produce a double-strand break (DSB), so their use as a pair should thus generate a large deletion. The protospacers were cloned separately into pX458 plasmids encoding the sgRNA scaffold and Cas9.

### 2.2. CRISPR-Mediated Large Deletions in the GAS5 Gene Are Present in Only One Allele and Result in the Downregulation of the Target lncRNA

**Figure 4:**
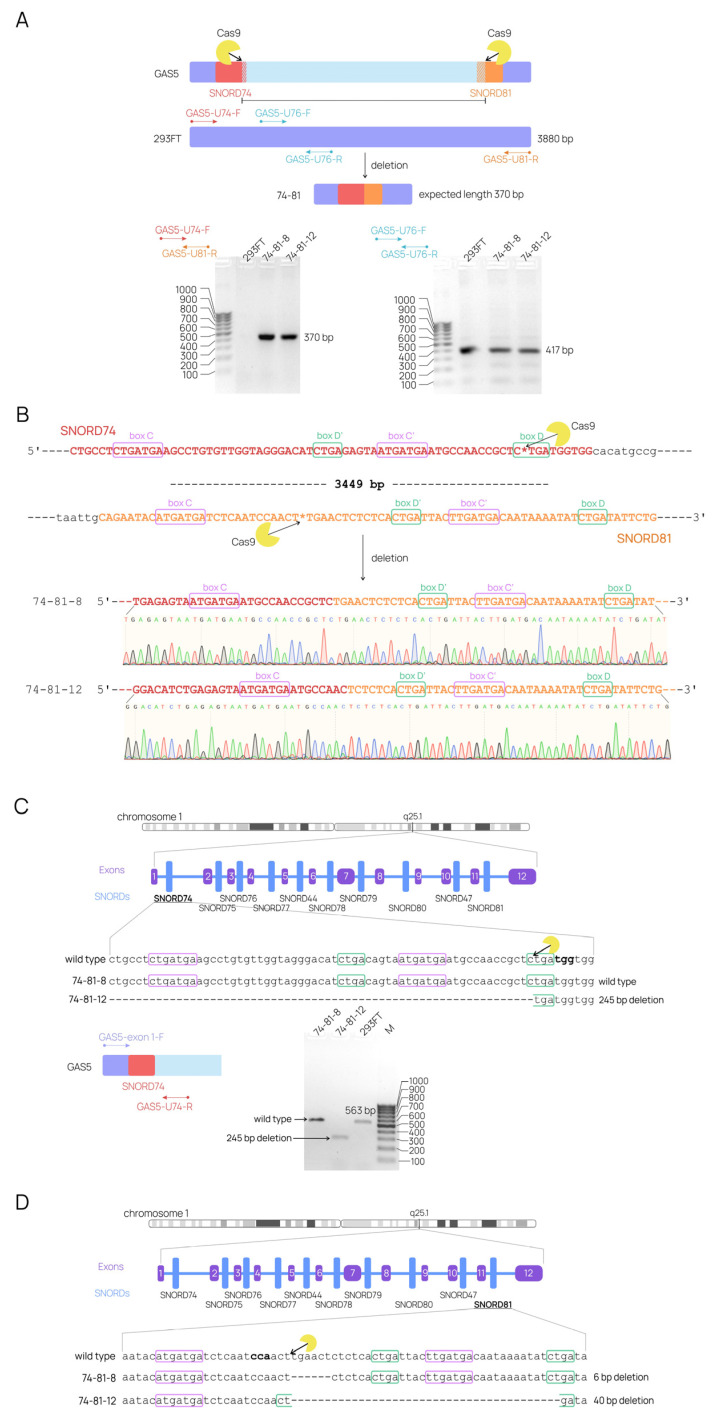
CRISPR/Cas9-induced genome alterations. (**A**) PCR-based screening for the expected deletion “74-81”. PCR products were analyzed using electrophoresis in 1.5% agarose gel (performed independently three times). The region of intron 4 covering the *SNORD76* gene was amplified to test for the presence of a second allele without the large deletion. Products were analyzed in 1.5% agarose gel (performed independently three times). (**B**) Sanger sequencing of the deletion region was performed to confirm the formation of a novel junction. Asterisks denote the target DSB sites. (**C**,**D**) Sanger sequencing of the *GAS5* regions covering target *SNORD* genes was performed to identify point mutations in the second alleles of *SNORD74* (**C**) and *SNORD81* (**D**). PCR products of the target *SNORD74* region were analyzed in a 1.5% agarose gel to visualize the short product from the 74-81-12 cell line.

In this experiment, 293FT cells were transfected with a pair of plasmids. GFP-positive cells were then sorted, and viable monoclonal cell lines carrying deletions of interest in the target gene were selected. Two of the cell lines had large deletions covering the region from *SNORD74* to *SNORD81* (clones 74-81-8 and 74-81-12; Figure 4A). The deletion was detected by PCR using flanking primers, with further analysis on agarose gel.

To test whether the deletion occurred in one or both alleles, another PCR was performed using a set of primers (GAS5-U76-F/R) flanking the region within the deletion area (Figure 4A). The wild-type product was present in all of the obtained cell lines, indicating that the modified cell lines were heterozygous, with no deletion in the second allele. Sanger sequencing confirmed the formation of the desired junction (Figure 4B).

However, it was still unclear whether any point mutations occurred in the second allele of the target *SNORD* genes. To test for point mutations, we amplified the target *SNORD* gene region and performed Sanger sequencing, which was followed by data analysis using TIDE and ICE tools (Figure 4C,D). In the 74-81-8 cell line, the second *SNORD74* allele remained unaltered, while the *SNORD81* gene carried a 6 bp deletion partially covering the complementarity region. In the 74-81-12 cell line, mutations were observed in both *SNORD74* and *SNORD81*. A 245 bp deletion upstream of the predicted DSB was present in the *SNORD74* gene, resulting in almost complete loss of the gene. In *SNORD81,* a 40 bp deletion covering the guide region and the D′- and C′-boxes was detected. Thus, two monoclonal cell lines were obtained, each carrying the desirable large deletion in one *GAS5* allele and either a point mutation or no mutation in a target *SNORD* gene in the second allele.

**Figure 5:**
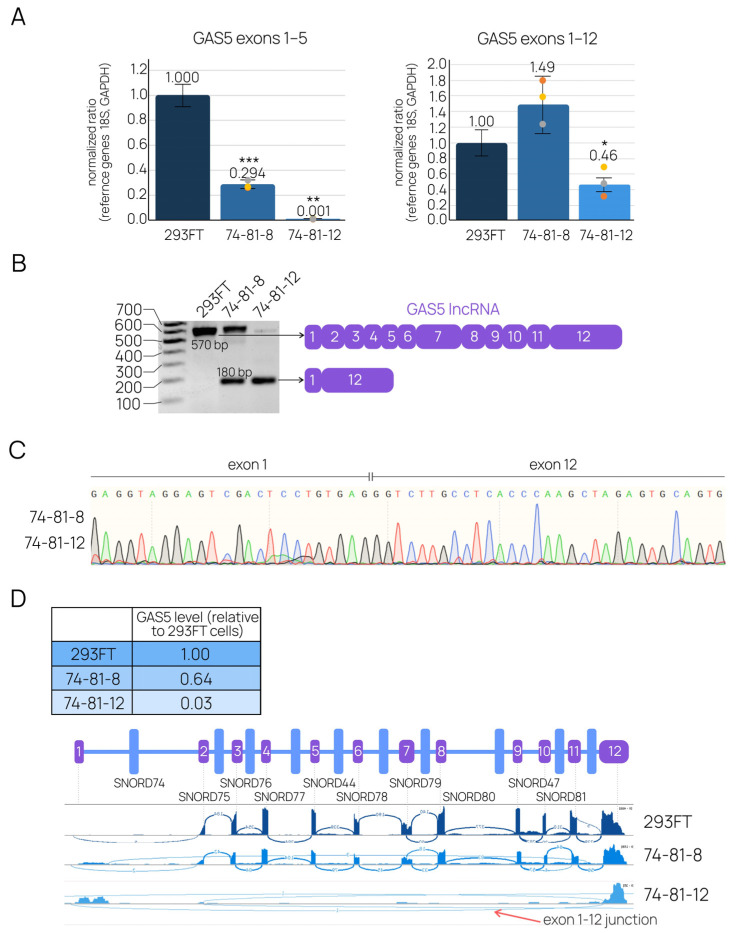
Analysis of mature GAS5 lncRNA in the modified cell lines. (**A**) Real-time quantitative RT-PCR, using primer pairs covering various GAS5 regions, was performed to measure the level of lncRNA. Relative ratios were additionally normalized to the level of the product in control cells. Data are presented as means ± standard deviation (SD) from three independent experiments performed in triplicate, with colored dots marking individual data points. The differences were considered statistically significant at *p*-values < 0.05 (*), < 0.01 (**), and *p* < 0.001 (***). (**B**) RT-PCR products were analyzed with 1.5% agarose gel electrophoresis (performed independently three times). The lengths of the products corresponded to the predicted lengths of mature lncRNA GAS5 variants, including novel variants. (**C**) Sanger sequencing of the shortened GAS5 confirmed the formation of the novel exon-exon junction. (**D**) RNA-Seq of the polyA-RNA fraction demonstrated the decrease in the total level of GAS5 lncRNA, as well as a partial loss of exons in modified cell lines and the formation of novel exon-exon junctions, as seen in the Sashimi plot.

We next sought to assess the levels of the target host-gene lncRNA GAS5, as well as snoRNAs encoded by genes located within the deletion area. qRT-PCR with primer pairs covering various regions of GAS5 lncRNA demonstrated a decrease in levels of the mature RNA in both “74-81” cell lines (Figure 5A). The analysis of qRT-PCR products in agarose gel revealed both wild-type and shortened variants (Figure 5B). Subsequent Sanger sequencing of the shortened product showed that mature lncRNA forms from the depleted allele with a non-canonical exon 1-12 junction (Figure 5C). Whole-transcriptome RNA-Seq analysis of the obtained clones supported these findings (Figure 5D, Supplementary Figure S1).

**Figure 6:**
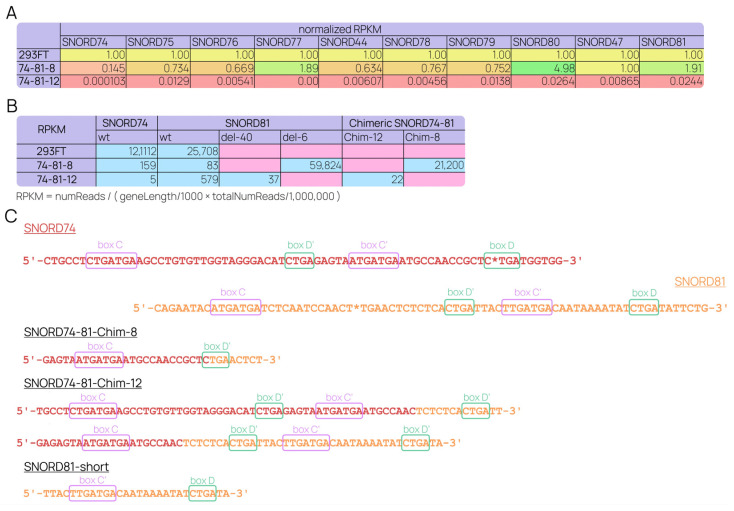
The analysis of snoRNA expression in modified cell lines. (**A**) Levels of ten *GAS5*-encoded box-C/D-snoRNAs were assessed by RNA-Seq of the small RNA fraction. Normalized-to-control ratios are presented. (**B**) RNA-Seq revealed the formation of mature chimeric SNORD74-81 forms. RPKMs are compared to the expression of the wild-type target snoRNAs in the control cell line. Del-40 and del-6 refer to mature SNORD81 shortened by 40 nt and 6 nt, respectively. (**C**) Sequences of chimeric and short SNORDs retrieved from the RNA-Seq data. Asterisks denote the expected sites for CRISPR-induced DSBs.

RNA-Seq data for the small RNA fraction demonstrated the downregulation of snoRNAs encoded in the introns within the deletion region (Figure 6A). qRT-PCR using primers covering *GAS5*-encoded snoRNA ends supported these findings (Supplementary Figure S2). Notably, mature chimeric U74-U81 snoRNAs were present in small quantities (Figure 6B,C). These novel forms were short and contained the canonical box pairs. The 74-81-8 cell line expressed a mutant SNORD81 lacking 6 nt in the complementary region (SNORD81-del-6) as a result of maturation of the second *GAS5* allele without the continuous “74-81” deletion. It is worth noting that this mutation seems to stabilize the functional snoRNA structure, as the total SNORD81 was upregulated. In the 74-81-12 cell line, SNORD81-del-40, which was shortened by 40 nt, was also identified as a mature small RNA. However, its expression was particularly low, likely due to the significant alterations in its structure. Interestingly, a wild-type SNORD81 was detected in the 74-81-12 cell line (Figure 6B) as a short, 28-nt snoRNA-derived RNA (“SNORD81-short” sdRNA; Figure 6C).

The results obtained demonstrate that CRISPR/Cas9-mediated depletion of *GAS5* can be produced in only one allele, and stable human cell lines exhibit downregulation of target lncRNA and snoRNAs from the region with the deletion. Remarkably, the downregulation patterns of snoRNAs differ between “74-81” cell lines, even though both carry a large *GAS5* deletion in one allele. The 74-81-12 clone demonstrates the complete downregulation of all ten snoRNAs and the mature GAS5 lncRNA. In contrast, the 74-81-8 cell line does not exhibit this pattern, indicating that it is not the allele with the deletion that influences GAS5 lncRNA maturation, but rather the second allele. Rigorous analysis of the CRISPR/Cas9-mediated genome alterations suggests that such diverse downregulation patterns can be caused by point mutations in target *SNORD* genes within the second *GAS5* allele. To test this hypothesis, we created and analyzed human cell lines with individual *SNORD74* or *SNORD81* mutations.

### 2.3. Individual SNORD Mutations Affect GAS5 and snoRNAs Maturation

It has previously been shown that mutations in individual *GAS5*-encoded *SNORD* genes affect the splicing of GAS5, potentially via an m6A-dependent mechanism [67]. In this sense, the above-mentioned human cell lines that carry a large *GAS5* deletion provide quite an interesting and promising model, with one *GAS5* allele being knocked out and the other bearing point mutations in *SNORD* genes. To investigate how specific mutations influence GAS5 maturation, we obtained human cell lines with mutations in individual *SNORD* genes, namely *SNORD74* and *SNORD81*. One of these cell cultures has already been described [67].

**Figure 7:**
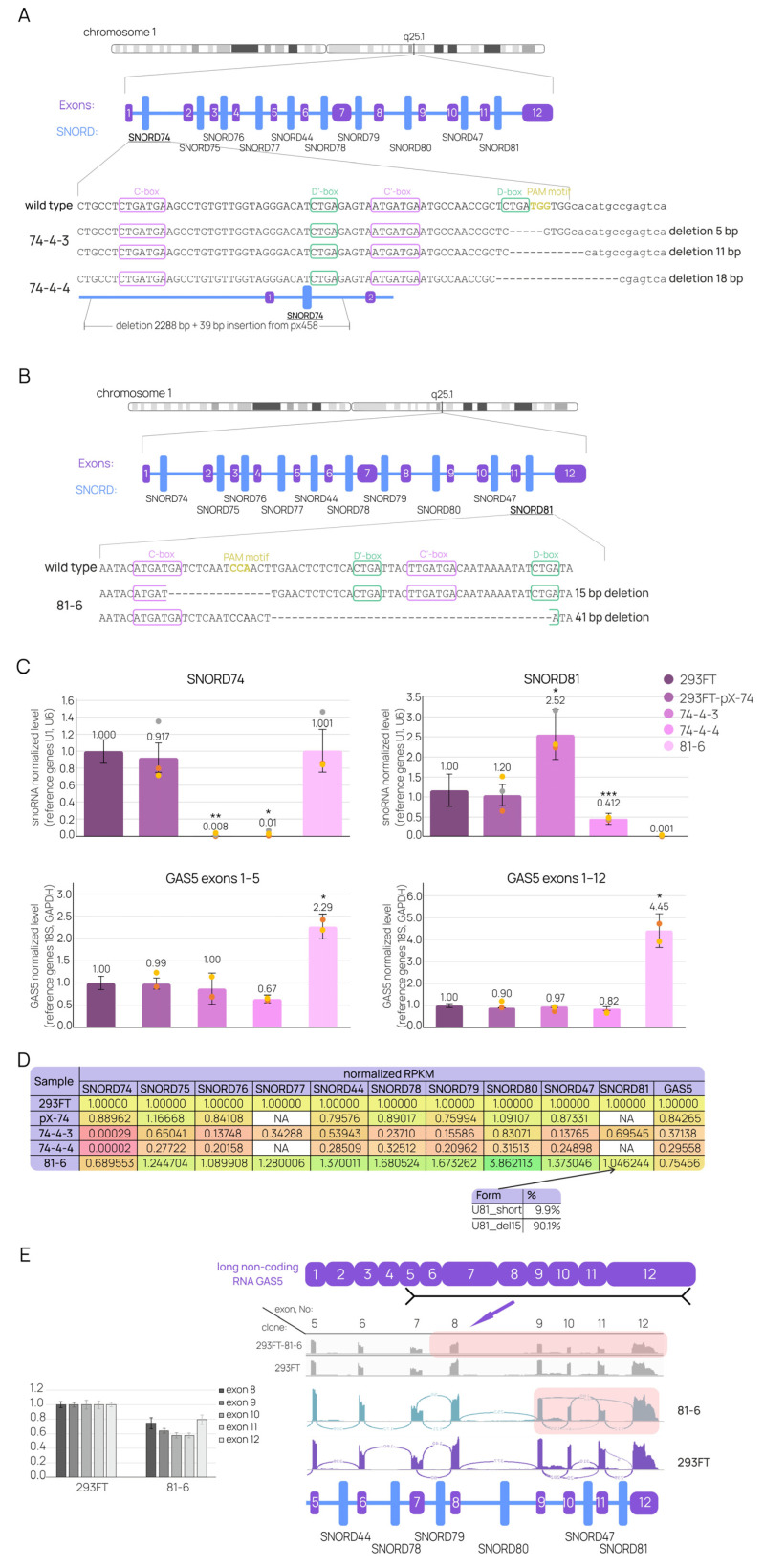
Individual snoRNA-knockout cell lines were utilized as a model for studying GAS5 maturation and splicing. Single-cell clones were obtained from 293FT cells. CRISPR/Cas9-induced point mutations in *SNORD74* (**A**) or *SNORD81* (**B**) were analyzed using Sanger sequencing of target genome regions. (**C**) The levels of mature target SNORDs and mature lncRNA GAS5 were assessed by quantitative RT-PCR. *U1* and *U6* snRNA genes were used as reference genes, as were *18S* and *GAPDH*. Data are presented as means ± standard deviation (SD) from three (for SNORDs) and two (for GAS5) independent experiments performed in triplicate, with colored dots marking individual data points. The differences were considered statistically significant at *p*-values < 0.05 (*), <0.01 (**), and <0.001 (***). (**D**) RNA-Seq data revealed alterations in the levels of all 10 *GAS5*-encoded SNORDs and GAS5 lncRNA. The distribution of mature mutant SNORD81 forms was also assessed. Data are presented as RPKM normalized to the control 293FT cell line. “NA” refers to the quantification that failed to provide the data. (**E**) The analysis of splicing identified a novel GAS5 variant expressed by the 81-6 cell line. The relative decrease in the levels of exons 8-12 is presented alongside the Sashimi plot for the same region, indicating the formation of a novel exon-exon junction.

In this experiment, 293FT cells were subjected to a routine CRISPR/Cas9 editing protocol using individual pX458 plasmids to generate large *GAS5* deletions (Figure 3). The obtained cell lines were analyzed for mutations in *SNORD* genes using Sanger sequencing of PCR products amplified with primer pairs flanking target *SNORD* regions. Two cell lines with *SNORD74* mutations and one cell line with a *SNORD81* mutation were selected for further analysis (Figure 7A,B).

In cell line 74-4-3, one *SNORD74* allele carried a 5 bp deletion and the second contained an 11 bp deletion, with both mutations covering the 3′-end and thus leading to the loss of the D-box [67]. Cell line 74-4-4 was heterozygous as well. One of its *SNORD74* alleles contained an 18 bp deletion covering the 3′-end, which resulted in the loss of D-box. The second allele contained a large deletion that led to the loss of the 5′-end of *GAS5* (including exon 1 and the greater part of the first intron), as well as an insertion of a small sequence derived from the pX458 vector, probably as a result of recombination during repair (Figure 7A).

Cell line 81-6 was heterozygous, with both *SNORD81* mutations being deletions. One allele contained a 15 bp deletion partially covering the C-box and its 3′-adjacent area. The second *SNORD81* allele of the 81-6 cell line carried a 41 bp deletion covering the D- and C′/D′-boxes, as well as the functional guide region (Figure 7B).

A “reference” cell line pX-74 was transfected with the plasmid pX458-74 but had no mutations in the *SNORD74* gene. It was included in the analysis to eliminate any effects caused by the plasmid treatment or CRISPR/Cas9 activity.

Selected cell lines were further analyzed for changes in the expression of *GAS5*-encoded SNORDs and lncRNA. qRT-PCR of SNORD74 in the 74-4 cell lines demonstrated a significant decrease, up to complete downregulation, likely due to the 3′-end deletions causing structural perturbations. qRT-PCR of SNORD81 in the 81-6 cell line revealed no differences in the level of target snoRNA, which indicates that mutations do not prevent the maturation of the mutant snoRNA. qRT-PCR of mature GAS5 lncRNA demonstrated a slight decrease in the levels of the transcripts in the 74-4 cell lines and an upregulation in the 81-6 cell line (Figure 7C).

RNA-Seq results supported some of these findings, adding new data (Figure 7D). The levels of target snoRNAs were confirmed by two independent methods, and the distribution of different mutant SNORD81 forms in the 81-6 cell line was estimated by RNA-Seq. The form constituting the greatest percentage of mature SNORD81 was found to be the 15-nt depleted form (U81_del15—90.1%). Interestingly, 9.9% of the mature SNORD81 was made up of a shortened SNORD81 (U81_short), which had already been observed as the 28 nt “short” sdRNA in the control cell line and partially *GAS5*-depleted cells (Figure 6B). The 15 bp depleted allele appeared to produce this form. The levels of other *GAS5*-encoded SNORDs were assessed as well (Figure 7D). Interestingly, while *SNORD81* mutations in the 81-6 cell line did not affect the maturation of snoRNAs, *SNORD74* mutations in the 74-4 cell lines led to varying degrees of downregulation in all 10 snoRNAs. Additionally, levels of the mature GAS5 lncRNA were confirmed to be decreased to different extents in the 74-4 cell lines and the 81-6 cell line.

Overall, the upregulation of GAS5 in the 81-6 cell line, as indicated by qRT-PCR, might actually imply the formation of novel splice variants, and further analysis of RNA-Seq data supported this hypothesis. While *SNORD74* mutations had no effect on GAS5 splicing in the 74-4 cell lines, *SNORD81* mutations in the 81-6 cell line seemed to cause the formation of a novel variant lacking exons 10 and 11 and demonstrating a non-canonical exon9-exon12 junction (Figure 7E).

Taken together, these data confirm that *SNORD*s contain regulatory regions affecting the maturation and splicing of GAS5. Expression patterns in the 81-6 cell line support the existing hypothesis regarding m6A-dependent GAS5 splicing. Results obtained from cell lines carrying the individual *SNORD74* knockout indicate that *SNORD74* contains a specific regulatory region modulating maturation of the GAS5 precursor.

### 2.4. Differential Gene-Expression Analysis Sheds Light on the Molecular Mechanisms behind the Functioning of GAS5 lncRNA in Human Cells

**Figure 8:**
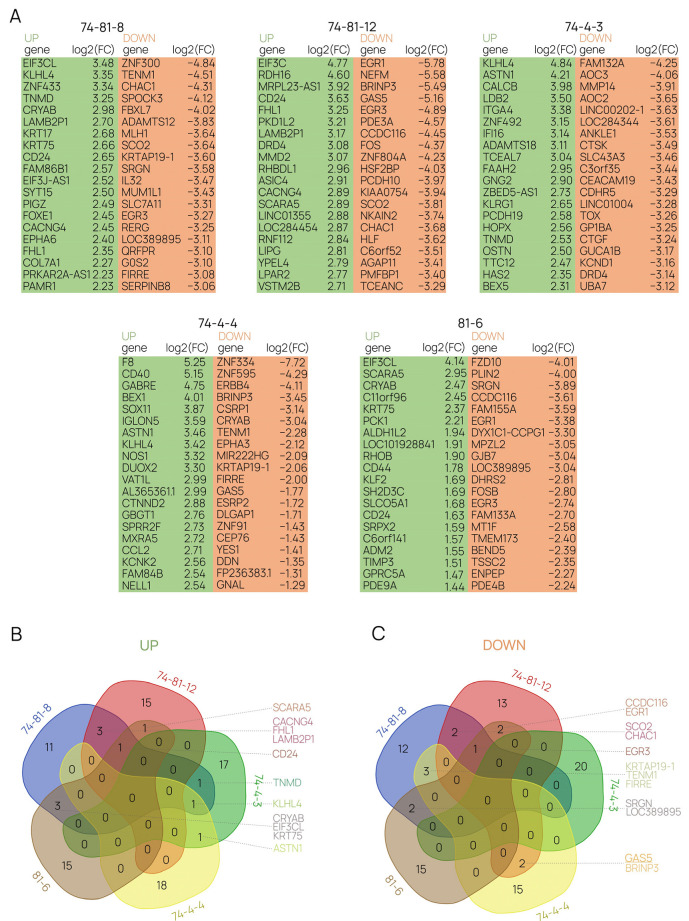
Differential expression analysis. (**A**) The top-20 upregulated (“UP”, green) and downregulated (“DOWN”, orange) DEGs were identified for each cell line obtained in the study. (**B**,**C**) The top DEGs were used to generate Venn diagrams of the upregulated and downregulated DEGs in 293FT cell lines with varying degrees of GAS5 suppression.

Based on analysis of RNA-Seq data, we created lists of differentially expressed genes (DEGs) for each of the above-mentioned cell lines (Materials and Methods, Section 4.7). Top-20 DEGs (Figure 8A) displaying statistically significant (q-value < 0.05) upregulation (log2(FC) > 2, where FC stands for fold change) or downregulation (log2(FC) < −2) were analyzed with DAVID and ENRICHR tools (accessed on 7 December 2023) [68,69,70,71]. Resulting clusters, annotations, processes, and pathways were filtered for statistical significance (*p*-value < 0.05; Tables S1 and S2). Filtered DEGs were also used to generate Venn diagrams in order to demonstrate the putative similarity in the upregulation and downregulation patterns for all of the obtained cell lines (Figure 8B,C).

Upregulated DEGs displayed no direct association with any major cellular processes or pathways in any of the clones, with most annotations relating to cell adhesion, signal transduction, and cell-membrane structures (Table S1). The analysis of associations with transcription factors (TFs), on the other hand, yielded interesting findings. Both “74-81” cell lines and the 74-4-4 clone demonstrate a connection between the most-upregulated DEGs and SUZ12. The 81-6 clone DEGs demonstrate a relationship to SOX2 and EZH2. The downregulated DEGs in the described clones provided a slightly clearer functional picture. The annotations ranged from signal transduction and extracellular regions to DNA binding and transcription regulation (Table S2). Cell lines “74-81”, 74-4-4, and 81-6 stood out as those in which the downregulated DEGs demonstrated associations with transcriptional gene networks, regulation of apoptosis and TFs CEBPB, SUZ12, NELFE, SRF, NR2F2, EZH2. Venn diagrams for both gene pools (“UP” and “DOWN”) revealed that there is not a large number of intersecting DEGs (Figure 8B,C).

## 3. Discussion

Our study demonstrated that multiple snoRNA and GAS5 knockouts in human cells can be generated using CRISPR/Cas9. The efficient and almost complete depletion of the host gene was accomplished by targeting two terminal introns in the *SNORD* regions simultaneously. This approach enabled the generation of human cell lines with varying downregulation of both the wild-type *GAS5*-encoded snoRNAs and GAS5 lncRNA itself.

Interestingly, only heterozygous cell lines bearing a continuous deletion in a single *GAS5* allele were obtained. This result might indicate that a complete loss of the *GAS5* gene cannot be achieved in human cells due to its crucial role as a snoRNA host in the genome. Nonetheless, the question of whether GAS5 RNA itself is essential remains open because viable cell lines with GAS5 knockdown have been obtained previously [21,72,73,74,75,76,77]. Given the fact that the experiments on siRNA- or shRNA-mediated knockdown are typically carried out in order to investigate the tumor-suppressing role of GAS5 in varying cancer types and result in the partial downregulation of GAS5 lncRNA (to the levels comparable with those in cell lines “74-4” and 74-81-8) we briefly compared the strategies and the effects detected.

The differential-expression analysis supports the observation regarding the viability of *GAS5*-depleted cells, as there was no drastic activation of pathways associated with cell death; however, a few remarks can be made. First, the association of downregulated DEGs with the transcriptional activation and, most importantly, the regulation of apoptosis is consistent with the existing data showing that GAS5 acts as a tumor suppressor through p53-induced apoptosis and downregulation of E2F1 [75,78]. Second, both up- and down-regulated DEGs were found to be connected with SUZ12 transcription factor, which is known to be a part of polycomb repressive complex 2 (PRC2) (Tables S1 and S2). GAS5 is able to epigenetically suppress gene expression through recruitment of another component of PRC2—EZH2, which provides H3 lysine 27 trimethylation (H3K27me3) [79,80,81]. On the other hand, GAS5 overexpression was shown to increase the amount of PRC2 in glioma cells, with the direct binding of GAS5 to EZH2 resulting in reduced promoter methylation and the induction of miR-424 expression [82]. Thus, the observed upregulation and downregulation of genes associated with SUZ12 in the obtained human cell lines support the idea of a tight connection between GAS5 and PRC2. While we were not able to discover any new interactions or pathways involving GAS5 lncRNA, we firmly believe that our results might be useful for future research on generating model cell lines with GAS5 downregulation as an alternative to knockdown techniques. We suggest that it is not only large deletions generated through the simultaneous editing of the first and last introns, but also local disruptions resulting in the loss of *SNORD74* gene that are effective for partial GAS5 knockdown.

Cell lines with large deletions in a *GAS5* allele provide a useful model for mechanistic studies on the maturation of the snoRNA host gene. Their single functional allele can be further modified to assess the influence of specific gene regions on the maturation process. The fact that the degree of downregulation varied among clones carrying a deletion in only one allele is quite peculiar. The second allele was modified less dramatically by two-point *SNORD* mutations in some cases; still, the maturation of GAS5 lncRNA and snoRNAs was partially or completely inhibited. Based on the results from the 74-81-8 cell line, the alterations in GAS5 do not depend on individual snoRNA levels. This result implies that it is not the transcription that is affected, but rather the maturation stages, particularly splicing. It is intriguing that specific point mutations in *SNORD* cause such an effect, thus suggesting that corresponding regions are responsible for the splicing and maturation tuning. While there are a number of studies devoted to non-canonical snoRNA functions, such as alternative mRNA splicing and its regulation or the modulation of cellular pathways with miRNA-like snoRNA derivatives, little is known about the universal mechanisms underlying the initial stages of host-gene maturation [26,83].

**Figure 9:**
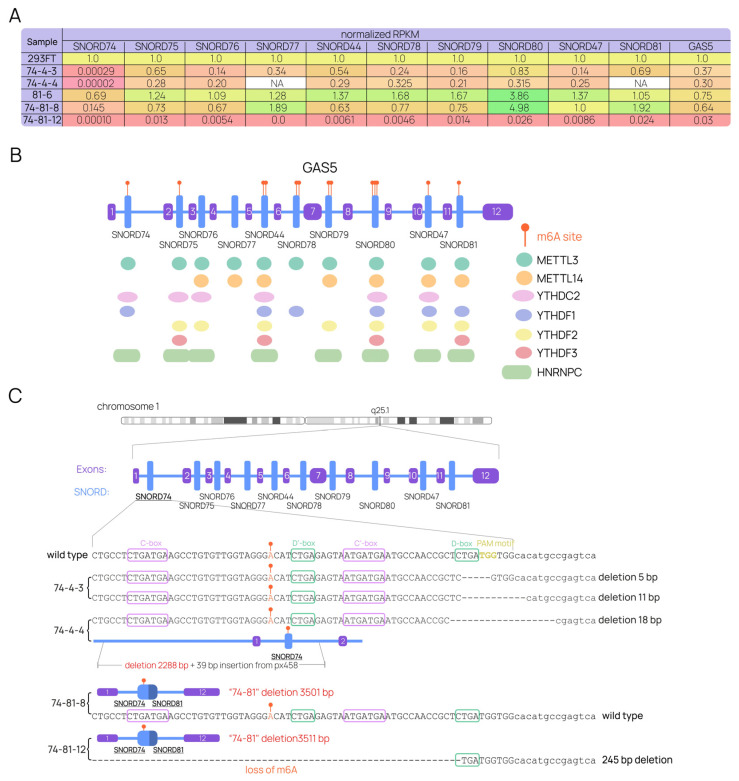
*SNORD*s contain regions regulating the maturation of the *GAS5* precursor transcript. (**A**) Combined RNA-Seq data on all human cell lines containing mutations in *SNORD74* or *SNORD81*. “NA” refers to the quantification that failed to provide the data. (**B**) GAS5-encoded SNORDs contain target nucleotides for m6A modifications, as well as binding sites for m6A-associated factors. (**C**) Small point mutations in *SNORD74* do not result in the loss of the nucleotide targeted by m6A modification; however, the 245 bp deletion observed in the 74-81-12 line depletes the modification area.

Taken together, our experimental data suggest that *SNORD74* is responsible for the fine-tuning of GAS5 splicing. Indeed, the cell line with individual *SNORD81* mutations displayed no significant decrease in the levels of GAS5-encoded snoRNAs or GAS5 lncRNA, whereas cell lines with individual *SNORD74* mutations showed downregulation of all 10 box-C/D-snoRNAs and GAS5 RNA (Figure 9A).

In our previous study, we proposed a mechanism by which GAS5 splicing is regulated [67], suggesting the involvement of N6-methyladenosine (m6A) in mRNA metabolism. Although most research focuses on the role of m6A in mRNA stability, degradation, and translation, recent studies have revealed the crosstalk between m6A modification and RNA splicing [60,84,85]. Early m6A deposition at splice junctions correlates with constitutive kinetics, whereas the presence of m6A within introns is associated with alternative splicing events, which highlights the role of m6A modification as a tool for fine-tuning maturation precursor [61]. Previously, we carried out a bioinformatical analysis using the POSTAR2 database to identify binding sites for the m6A-associated proteins within *GAS5* introns, specifically, in *SNORDs* (Figure 9B). We suggested that the loss of these sites upon CRISPR/Cas9 editing might disrupt the binding of m6A-interacting factors with the transcript and cause alternative splicing events, leading to the formation of novel GAS5 isoforms [67]. The present study supports this idea, as *SNORD81* contains binding sites for m6A-associated factors and the cell line with individual mutations in this gene produces a GAS5 lncRNA variant lacking exons 10 and 11 (Figure 7E).

We further analyzed data from RMBase v3.0 (RMBase v3.0: Decode the Landscape, Mechanisms and Functions of RNA Modifications) to identify target nucleotides for m6A modification in the *GAS5* structure. Eight *SNORD*s were found to contain such sites, including the gene of interest, *SNORD74* (Figure 9B). The target m6A-nucleotide is located in the canonical guide region, next to the nucleotide determining the 2′-O-methylation target in rRNA (Figure 9C). It is known that m6A modification in snoRNA might lead to the disruption of its structure and the loss of mature snoRNA in cells. Nevertheless, we can state that the SNORD74 site does not cause the same effects, as it is located outside of the K-turn motif [86]. Moreover, we suggest that the presence of m6A acts as a signal for the splicing machinery to “slow down” during the excision of the first intron [61]. This slowing allows for other splicing factors to become involved to support the proper maturation of the GAS5 transcript.

When studying cell lines containing any *SNORD74* mutations, several observations can be made (Figure 9C). The region that appears to regulate *GAS5* maturation is determined by its sequence: evidently, the loss of the greater part of *SNORD74* (as in the 74-81-12 line) leads to a drastic downregulation of GAS5 lncRNA and all 10 snoRNAs. In contrast, clones with mutations in the 3′-region of *SNORD74* not involving the m6A site directly (both 74-4 clones and the 74-81-8 line) do not demonstrate the same degree of decrease in RNA levels. Observed variations in RNA levels could result from the loss of the optimal secondary structure in the *SNORD74* transcript region, which is necessary for the correct interaction with m6A-associated proteins. Thus, the hypothesis regarding the m6A-dependency of GAS5 splicing regulation can be extended to include *SNORD74*. We propose that m6A modification in *SNORD74* is responsible for the maturation of the whole GAS5 RNA precursor, consequently determining the fate of the mature GAS5 lncRNA and intron-encoded snoRNAs. This hypothesis is supported by the maturation of a short GAS5 lncRNA comprised of exons 1 and 12 in cell lines “74-81” (Figure 5B–D). The continuous deletion of *GAS5* does not affect the m6A site in *SNORD74* (Figure 4B), thus allowing for the maturation of the *GAS5* precursor. While other m6A sites in *SNORDs* can be responsible for alternative splicing of GAS5, as seen in the 81-6 line, the localization of *SNORD74* specifically in the first intron makes it a convenient “switch”. We assume that such a mechanism might be implemented universally for other snoRNA host genes (*SNHG*s), as the database analysis revealed similar patterns of m6A-site deposition in their respective intronic *SNORD* genes. Analogous studies on the point editing of *SNHG*s introns, as well as generation of cell lines depleted of *SNHG*s in a similar manner, are promising as a foundation for the characterization of this novel epitranscriptomic mechanism.

The presence of novel short snoRNAs in the obtained cell lines also sparks significant interest (Figure 6B). We named these snoRNA variants “chimeric” (chisnoRNA), as they are derived from the fragments of two naturally occurring SNORDs. The formation of such snoRNAs indicates that it is possible to obtain human cell lines stably expressing novel non-natural snoRNAs. This finding is intriguing in terms of generating cell lines that express artificial snoRNAs to study the pathways regulating the expression of various mammalian genes, as well as to develop tools for its modulation. For example, one appealing strategy would be to make a cell line stably expressing the naturally occurring snoRNA with a modified guide region in order to re-target its activity at an RNA of choice.

## 4. Materials and Methods

### 4.1. Materials

Plasmids were generated using *BstV2I* restriction endonuclease (SibEnzyme, Novosibirsk, Russia) and T4 DNA ligase (Thermo Fisher Scientific, Waltham, MA, USA). Routine amplification and clone testing were performed using the BioMaster HS-Taq PCR-Color (2×) mix (Biolabmix Ltd., Novosibirsk, Russia). Oligonucleotide primers were synthesized by the Laboratory of Synthetic Biology (ICBFM SB RAS) or Biosset Ltd. PCR products were isolated using the DR Kit (Biolabmix Ltd., Novosibirsk, Russia). Sanger sequencing was carried out using the BigDye™ Terminator v3.1 Cycle Sequencing Kit (Thermo Fisher Scientific), and products were further analyzed on the ABI 3130XL Genetic Analyser at the SB RAS Genomics Core Facility. Plasmid constructs were isolated with the Plasmid Mini and EndoFree Plasmid Maxi kits (both QIAGEN GmbH, Hilden, Germany). Genomic DNA was isolated using the Genomic DNA Isolation Kit (Biolabmix Ltd., Novosibirsk, Russia). Total RNA and small RNA fractions were isolated using the LIRA reagent and the LRU RNA Isolation Kit (Biolabmix Ltd., Novosibirsk, Russia). Quantitative RT-PCR was performed using the BioMaster RT-PCR SYBR Blue reaction mix (Biolabmix Ltd., Novosibirsk, Russia) and a LightCycler 96 thermocycler (Roche, Switzerland).

### 4.2. Generation of CRISPR/Cas9 Constructs

Two protospacer sequences were selected for the specific cleavage of snoRNA genes encoded within *GAS5* introns. The protospacers were tested for possible off-target effects using the Benchling tool (Benchling, RRID:SCR_013955). Plasmid pSpCas9(BB)-2A-GFP (pX458; Addgene, #48138) was used as an expression vector [87]. Guide-determining oligonucleotides for the *SNORD74* target (74-4-t 5′-CACCGATGAATGCCAACCGCTCTGA-3′ and 74-4-b 5′-AAACTCAGAGCGGTTGGCATTCATC-3′) and the *SNORD81* target (81-1-t 5′-CACCGATCAGTGAGAGAGTTCAATG-3′ and 81-1-b 5′-AAACACTTGAACTCTCTCACTGATC-3′) were annealed and cloned into the pX458 vector using *BstV2I* restriction endonuclease (SibEnzyme, Russia) and T4 DNA ligase (Thermo Fisher Scientific) according to the standard protocol [87]. Competent TOP10 *E. coli* cells were transformed with the obtained constructs, spread onto LB agar plates supplemented with ampicillin at a concentration of 10 mg/mL, and incubated overnight at 37 °C. Colonies containing the pX458 plasmid with sgRNA insertion were selected by colony PCR and Sanger sequencing, and CRISPR/Cas9 expression vectors were then isolated using the EndoFree Plasmid Maxi Kit (Qiagen).

### 4.3. Cell Culture and Transfection

A human 293FT cell line (Thermo Fisher Scientific) was used in the study. Cells were maintained in a 1:1 mixture of Dulbecco’s Modified Eagle’s Medium and Ham’s F12 media (DMEM/F12, Gibco) supplemented with 10% fetal bovine serum (FBS, Gibco) and 1% MEM NEAA, sodium pyruvate, GlutaMax, and antibiotic–antimycotic (all Gibco) at 37 °C with 5% CO_2_. Cells were seeded in 6-well plates at a density of ~0.4 × 10^6^ cells per well 24 h prior to transfection. Transfection of the cells with 2 µg of a single expression vector or the pair of expression vectors in a 1:1 ratio was performed in the serum-free full DMEM/F12 medium using the Lipofectamine 3000 reagent (Thermo Fisher Scientific) according to the manufacturer’s instructions. Cells transfected with the pX458 plasmid carrying a sgRNA sequence lacking the spacer region were used as the transfection control.

### 4.4. Individual Clone Selection and the Identification of Mutations

GFP-positive cells were selected and seeded (1 cell per well) into a 96-well plate by FACS (SH3800 Cell Sorter, Sony Biotechnology) 48 h after transfection. After they reached ~80–90% confluency, cells were divided equally between two 96-well plates. One of the plates was then used for the PCR-based mutation screening. Genomic DNA was isolated using a genomic DNA isolation kit (Biolabmix, Novosibirsk, Russia). To test for large deletions in the *GAS5* gene, PCR was performed using sets of specific primers (GAS5-U74-F 5′-AGCCTTTGTCTGCTAAGGTCA-3′ and GAS5-U74-R 5′-GTTGCCATTAACCGATGTCGA-3′ for the *SNORD74* region; GAS5-U81-F 5′-CTGAGAAGGAAATTGAGTAGG-3′ and GAS5-U81-R 5′-TCAAAGGCCACTGCACTCTAG-3′ for the *SNORD81* region). The primers flanked the expected deletion region (GAS5-U74-F and GAS5-U81-R) or an individual target *SNORD* gene. PCR products were then analyzed in a 1.5% agarose gel. To test whether one or both alleles contained the large deletion, PCR was performed using primers (GAS5-U76-F 5′-TGGTCTCAGCCTGTGATGCT-3′ and GAS5-U76-R 5′-CTGTGTGCCAATGGCTTGAG-3′) flanking the *SNORD76* region within the area of deletion, and the products were subsequently analyzed in a 1.5% agarose gel. Heterozygous modified cell lines and individual *SNORD*-knockout lines were analyzed for the presence of point mutations in the target genes. PCR products were isolated and subjected to Sanger sequencing. Mutations were analyzed using the TIDE and ICE assays [88] (Synthego Performance Analysis, ICE Analysis. 2019. v3.0. Synthego).

### 4.5. Isolation of Total Cell RNA

Total RNA and small RNA (<200 nucleotide length) fractions were extracted from cells using the phenol-chloroform method followed by isolation on absorption columns using the LRU-100-50 kit (Biolabmix, Novosibirsk, Russia). Following RNA elution in nuclease-free water, RNA concentration was assessed using the Qubit RNA HS Assay Kit (Thermo Fisher Scientific, USA) with Qubit 2 fluorometer (Thermo Fisher Scientific, USA). The quality of total RNA, expressed as the RNA Integrity Number (RIN), was determined using Bioanalyzer 2100 (Agilent, Santa Clara, CA, USA) and the Agilent RNA Pico 6000 Kit (Agilent, USA) [89]. A threshold RIN value greater than 7.0 was taken as the cut-off point for the transition to library preparation. The efficiency of enrichment for small RNAs and their length distribution were evaluated using 1.5% TAE-agarose gel and the Bioanalyzer 2100 instrument with the Agilent Small RNA kit (Agilent, USA). For sequencing, library preparation, and RT-qPCR analysis, solutions of extracted total RNA and small RNA were treated with DNase I (Thermo Fisher Scientific, USA) to remove DNA.

### 4.6. Library Preparation and Sequencing

A total of 22 cDNA libraries (11 for small RNAs and 11 for poly(A)+ RNAs) were prepared from two biological replicates. cDNA libraries were constructed according to a standard protocol using the NEBNext Multiplex Small RNA Library Prep Kit for Illumina (New England Biolabs, UK) for the small-RNA fraction, the NEBNext Ultra II Directional RNA library preparation kit for Illumina (New England Biolabs, UK), and the NEBNext mRNA Magnetic Isolation Module (New England Biolabs, UK) for the poly(A)+ RNA fraction. Fragment size distributions were analyzed using a Bioanalyzer 2100 (Agilent, USA) with the Agilent High Sensitivity DNA Kit (Agilent, USA), and the fragments were quantified with the Qubit 2 fluorometer (Thermo Fisher Scientific, USA) and the Qubit DNA HS Assay Kit (Thermo Fisher Scientific, USA). Libraries were sequenced on the Illumina NextSeq 500 instrument in 75-base-pair-single-end mode (NextSeq 500/550 High Output v2.5 Kit (Illumina, San Diego, CA, USA)). Binary Base Call files provided by the Illumina Real-Time Analysis RTCA Software version 1.2 software (ACEA Bioscience, United States) were de-multiplexed and converted into FASTQ format using bcl2fastq2 Conversion Software (v2.20). The construction of cDNA libraries and massive parallel sequencing were carried out at the Institute of Fundamental Medicine and Biology, Kazan Federal University (Kazan, Russia).

### 4.7. RNA-Seq and Differential Expression Analysis

The raw data were saved as FASTQ files. Quality-control analysis of the raw and trimmed reads was performed using FastQC (v0.11.9) and MultiQC (v1.9) [90,91]. Trimming of the adapter content and quality trimming were performed using fastp (v0.21.0) [92]. The reads complementary to the ribosomal RNA were filtered out using SortMeRNA (v2.1b) [93]. The filtered reads were aligned to the human genome (GRCh37) in STAR (v2.7.7a) [94]. The CuffDiff program was used for the comparative analysis of the differential gene and small-RNA expression [95]. Sashimi plots for the RNA-Seq analyses of the isoform expression were generated by IGV [96,97]. RNA-seq data were deposited in the ArrayExpress database at EMBL-EBI (https://www.ebi.ac.uk/biostudies/arrayexpress, accessed 27 October 2023) under the accession number E-MTAB-13515 [98].

### 4.8. Real-Time RT-PCR

Prior to quantitative RT-PCR, total RNA and small-RNA fractions were isolated from cells and treated with DNase I (Thermo Fisher Scientific), as mentioned in Section 4.5. After the concentration measurement, quantitative RT-PCR was performed.

To assess the total level of GAS5-encoded snoRNAs, the following primers were used: U74 RNA (U74-F 5′-CTGCCTCTGATGAAGCCTGTG-3′ and U74-R 5′-GAGCGGTTGGCATTCATC-3′); U75 RNA (U75-F 5′-AGCCTGTGATGCTTTAAGAG-3′ and U75-R 5′-AGCCTCAGAATAGAATTTCAG-3′); U76 RNA (U76-F 5′-TGCCACAATGATGACAGT-3′ and U76-R 5′-GCCTCAGTTAAGATAATGGTG-3′); U77 RNA (U77-F 5′-AGATACTATGATGGTTGC-3′ and U77-R 5′-GATACATCAGACAGATAG-3′); U44 RNA (U44-F 5′-CCTGGATGATGATAAGCA-3′ and U44-R 5′-AGTCAGTTAGAGCTAATTAAG-3′), U78 RNA (U78-F 5′-GTGTAATGATGTTGATCA-3′ and U78-R 5′-TTCTTCAGTGTTACCTTTG-3′), U79 RNA (U79-F 5′-CTGTTAGTGATGATTTAA-3′ and U79-R 5′-CTGTTTCAGTTTAAGATT-3′), U80 RNA (U80-F 5′-ACAATGATGATAACATAG-3′ and U80-R 5′-GATAGGAGCGAAAGACT-3′), U47 RNA (U47-F 5′-AACCAATGATGTAATGATTC-3′ and U47-R 5′-AACCTCAGAATCAAAATGG-3′), U81 RNA (U81-F 5′-CAGAATACATGATGATCTC-3′ and U81-R 5′-CAGAATATCAGATATTTTATTG-3′). The expression levels were presented as values normalized to the endogenous level of U1 (U1-F 5′-CAGGGGAGATACCATGATCACGAAG-3′ and U1-R 5′-CGCAGTCCCCCACTACCACAAAT-3′) and U6 (U6-F 5′-TCGCTTCGGCAGCACATATACTAAAAT-3′ and U6-R 5′-GAATTTGCGTGTCATCCTTGCG-3′) snRNAs.

To assess the level of mature GAS5 lncRNA, the following primers were used: GAS5 exons 1-5 (GAS5_exon1-F 5′-GAGGTAGGAGTCGACTCCTGTGA-3′ and GAS5_exon5-R 5′-CATTTCAACTTCCAGCTTTCTGT-3′); GAS5 exons 1-12 (GAS5_exon1-F and GAS5_exon12-R 5′-TTGGAGACACTGTTTTAATCTTCT-3′). The expression levels were presented as values normalized to the endogenous level of 18S rRNA (18S-F 5′-GATGGTAGTCGCCGTGCC-3′ and 18S-R 5′-GCCTGCTGCCTTCCTTGG-3′) and GAPDH mRNA (GAPDH-F 5′-GAAGATGGTGATGGGATTTC-3′ and GAPDH-R 5′-GAAGGTGAAGGTCGGAGT-3′). RT-PCR products resulting from the assessment of GAS5 levels were also analyzed in 1.5% agarose gel to detect the formation of potentially novel splice variants.

The mean values (± standard deviation, SD) from three independent experiments were calculated. Statistically significant differences were identified using paired Student’s *t*-test and denoted as *p*-values < 0.05 (*), <0.01 (**), or <0.001 (***).

## 5. Conclusions

This study characterized the maturation of the *GAS5* transcript in human cells. CRISPR/Cas9 editing of the first and the last intron simultaneously or independently allowed us to create a collection of human cell lines carrying various *GAS5* alleles. Cell lines with one depleted *GAS5* allele provided a promising model, allowing for the study of individual mutations in *SNORD* genes in order to shed light on the specifics of transcript maturation. Individual mutations in *SNORD81* were shown to promote the formation of a novel GAS5 lncRNA variant lacking exons 10 and 11. Mutations in *SNORD74* led to the loss of the target nucleotide for m6A modification, which hindered GAS5 lncRNA maturation and resulted in its downregulation and the downregulation of all of the intron-encoded box C/D snoRNAs. These findings suggest a novel, *SNORD*-dependent mechanism of snoRNA host-gene maturation that involves m6A-associated pathways.

## Data Availability

RNA-seq data have been deposited in the ArrayExpress database at EMBL-EBI (https://www.ebi.ac.uk/biostudies/arrayexpress, accessed 27 October 2023) under the accession number E-MTAB-13515.

## Supplementary Materials

The following supporting information can be downloaded at https://www.mdpi.com/article/10.3390/ijms242417621/s1.

## References

[1] Schneider C., King R.M., Philipson L. (1988). Genes Specifically Expressed at Growth Arrest of Mammalian Cells. Cell.

[2] Pickard M., Williams G. (2015). Molecular and Cellular Mechanisms of Action of Tumour Suppressor GAS5 LncRNA. Genes.

[3] Goustin A., Thepsuwan P., Kosir M., Lipovich L. (2019). The Growth-Arrest-Specific (GAS)-5 Long Non-Coding RNA: A Fascinating LncRNA Widely Expressed in Cancers. Noncoding RNA.

[4] Smith C.M., Steitz J.A. (1998). Classification of Gas5 as a Multi-Small-Nucleolar-RNA (SnoRNA) Host Gene and a Member of the 5′-Terminal Oligopyrimidine Gene Family Reveals Common Features of SnoRNA Host Genes. Mol. Cell Biol..

[5] Mourtada-Maarabouni M., Pickard M.R., Hedge V.L., Farzaneh F., Williams G.T. (2009). GAS5, a Non-Protein-Coding RNA, Controls Apoptosis and Is Downregulated in Breast Cancer. Oncogene.

[6] Li J., Huang H., Li Y., Li L., Hou W., You Z. (2016). Decreased Expression of Long Non-Coding RNA GAS5 Promotes Cell Proliferation, Migration and Invasion, and Indicates a Poor Prognosis in Ovarian Cancer. Oncol. Rep..

[7] Li G., Cai Y., Wang C., Huang M., Chen J. (2019). LncRNA GAS5 Regulates the Proliferation, Migration, Invasion and Apoptosis of Brain Glioma Cells through Targeting GSTM3 Expression. The Effect of LncRNA GAS5 on Glioma Cells. J. Neuro-Oncol..

[8] Kino T., Hurt D.E., Ichijo T., Nader N., Chrousos G.P. (2010). Noncoding RNA Gas5 Is a Growth Arrest- and Starvation-Associated Repressor of the Glucocorticoid Receptor. Sci. Signal.

[9] Hudson W.H., Pickard M.R., de Vera I.M.S., Kuiper E.G., Mourtada-Maarabouni M., Conn G.L., Kojetin D.J., Williams G.T., Ortlund E.A. (2014). Conserved Sequence-Specific LincRNA–Steroid Receptor Interactions Drive Transcriptional Repression and Direct Cell Fate. Nat. Commun..

[10] Zong Y., Zhang Y., Sun X., Xu T., Cheng X., Qin Y. (2019). MiR-221/222 Promote Tumor Growth and Suppress Apoptosis by Targeting LncRNA GAS5 in Breast Cancer. Biosci. Rep..

[11] Wang K., Li J., Xiong G., He G., Guan X., Yang K., Bai Y. (2018). Negative Regulation of LncRNA GAS5 by MiR-196a Inhibits Esophageal Squamous Cell Carcinoma Growth. Biochem. Biophys. Res. Commun..

[12] Zhang Z., Zhu Z., Watabe K., Zhang X., Bai C., Xu M., Wu F., Mo Y.Y. (2013). Negative Regulation of LncRNA GAS5 by MiR-21. Cell Death Differ..

[13] Shi Y., Parag S., Patel R., Lui A., Murr M., Cai J., Patel N.A. (2019). Stabilization of LncRNA GAS5 by a Small Molecule and Its Implications in Diabetic Adipocytes. Cell Chem. Biol..

[14] Li C., Lv Y., Shao C., Chen C., Zhang T., Wei Y., Fan H., Lv T., Liu H., Song Y. (2019). Tumor-Derived Exosomal LncRNA GAS5 as a Biomarker for Early-Stage Non-Small-Cell Lung Cancer Diagnosis. J. Cell Physiol..

[15] Dragomir M., Chen B., Calin G.A. (2018). Exosomal LncRNAs as New Players in Cell-to-Cell Communication. Transl. Cancer Res..

[16] Zhao J.H., Wang B., Wang X.H., Xu C.W. (2019). Effect of LncRNA GAS5 on the Apoptosis of Neurons via the Notch1 Signaling Pathway in Rats with Cerebral Infarction. Eur. Rev. Med. Pharmacol. Sci..

[17] Wu N., Zhang X., Bao Y., Yu H., Jia D., Ma C. (2019). Down-Regulation of GAS5 Ameliorates Myocardial Ischaemia/Reperfusion Injury via the MiR-335/ROCK1/AKT/GSK-3β Axis. J. Cell Mol. Med..

[18] Miao X., Liang A. (2019). Knockdown of Long Noncoding RNA GAS5 Attenuates H2O2-Induced Damage in Retinal Ganglion Cells through Upregulating MiR-124: Potential Role in Traumatic Brain Injury. J. Cell Biochem..

[19] Wang Y.-N.-Z., Shan K., Yao M.-D., Yao J., Wang J.-J., Li X., Liu B., Zhang Y.-Y., Ji Y., Jiang Q. (2016). Long Noncoding RNA-GAS5. Hypertension.

[20] Fang Y., Hu J.F., Wang Z.H., Zhang S.G., Zhang R.F., Sun L.M., Cui H.W., Yang F. (2018). GAS5 Promotes Podocyte Injury in Sepsis by Inhibiting PTEN Expression. Eur. Rev. Med. Pharmacol. Sci..

[21] Han X., Xu J., Chen Z., Li P., Zhao L., Tao J., Shen Y., Zhu S., Yu B., Zhu J. (2022). Gas5 Inhibition Promotes the Axon Regeneration in the Adult Mammalian Nervous System. Exp. Neurol..

[22] Jorjani H., Kehr S., Jedlinski D.J., Gumienny R., Hertel J., Stadler P.F., Zavolan M., Gruber A.R. (2016). An Updated Human SnoRNAome. Nucleic Acids Res..

[23] Watkins N.J., Dickmanns A., Luhrmann R. (2002). Conserved Stem II of the Box C/D Motif Is Essential for Nucleolar Localization and Is Required, Along with the 15.5K Protein, for the Hierarchical Assembly of the Box C/D SnoRNP. Mol. Cell Biol..

[24] Massenet S., Bertrand E., Verheggen C. (2017). Assembly and Trafficking of Box C/D and H/ACA SnoRNPs. RNA Biol..

[25] Bratkovič T., Božič J., Rogelj B. (2020). Functional Diversity of Small Nucleolar RNAs. Nucleic Acids Res..

[26] Baldini L., Charpentier B., Labialle S. (2021). Emerging Data on the Diversity of Molecular Mechanisms Involving C/D SnoRNAs. Noncoding RNA.

[27] Sharma S., Yang J., van Nues R., Watzinger P., Kötter P., Lafontaine D.L.J., Granneman S., Entian K.-D. (2017). Specialized Box C/D SnoRNPs Act as Antisense Guides to Target RNA Base Acetylation. PLoS Genet..

[28] Falaleeva M., Pages A., Matuszek Z., Hidmi S., Agranat-Tamir L., Korotkov K., Nevo Y., Eyras E., Sperling R., Stamm S. (2016). Dual Function of C/D Box Small Nucleolar RNAs in RRNA Modification and Alternative Pre-MRNA Splicing. Proc. Natl. Acad. Sci. USA.

[29] Scott M.S., Ono M., Yamada K., Endo A., Barton G.J., Lamond A.I. (2012). Human Box C/D SnoRNA Processing Conservation across Multiple Cell Types. Nucleic Acids Res..

[30] Stepanov G.A., Semenov D.V., Kuligina E.V., Koval O.A., Rabinov I.V., Kit Y.Y., Richter V.A. (2012). Analogues of Artificial Human Box C/D Small Nucleolar RNA As Regulators of Alternative Splicing of a Pre-MRNA Target. Acta Nat..

[31] Kishore S. (2006). The SnoRNA HBII-52 Regulates Alternative Splicing of the Serotonin Receptor 2C. Science.

[32] Lykke-Andersen S., Ardal B.K., Hollensen A.K., Damgaard C.K., Jensen T.H. (2018). Box C/D SnoRNP Autoregulation by a Cis-Acting SnoRNA in the NOP56 Pre-MRNA. Mol. Cell.

[33] Brandis K.A., Gale S., Jinn S., Langmade S.J., Dudley-Rucker N., Jiang H., Sidhu R., Ren A., Goldberg A., Schaffer J.E. (2013). Box C/D Small Nucleolar RNA (SnoRNA) U60 Regulates Intracellular Cholesterol Trafficking. J. Biol. Chem..

[34] Bergeron D., Fafard-Couture É, Scott M.S. (2020). Small Nucleolar RNAs: Continuing Identification of Novel Members and Increasing Diversity of Their Molecular Mechanisms of Action. Biochem. Soc. Trans..

[35] Youssef O.A., Safran S.A., Nakamura T., Nix D.A., Hotamisligil G.S., Bass B.L. (2015). Potential Role for SnoRNAs in PKR Activation during Metabolic Stress. Proc. Natl. Acad. Sci. USA.

[36] Lee J., Harris A.N., Holley C.L., Mahadevan J., Pyles K.D., Lavagnino Z., Scherrer D.E., Fujiwara H., Sidhu R., Zhang J. (2016). Rpl13a Small Nucleolar RNAs Regulate Systemic Glucose Metabolism. J. Clin. Investig..

[37] Michel C.I., Holley C.L., Scruggs B.S., Sidhu R., Brookheart R.T., Listenberger L.L., Behlke M.A., Ory D.S., Schaffer J.E. (2011). Small Nucleolar RNAs U32a, U33, and U35a Are Critical Mediators of Metabolic Stress. Cell Metab..

[38] Holley C.L., Li M.W., Scruggs B.S., Matkovich S.J., Ory D.S., Schaffer J.E. (2015). Cytosolic Accumulation of Small Nucleolar RNAs (SnoRNAs) Is Dynamically Regulated by NADPH Oxidase. J. Biol. Chem..

[39] Rogelj B., Hartmann C.E.A., Yeo C.H., Hunt S.P., Giese K.P. (2003). Contextual Fear Conditioning Regulates the Expression of Brain-Specific Small Nucleolar RNAs in Hippocampus. Eur. J. Neurosci..

[40] Li D., Zhang J., Wang M., Li X., Gong H., Tang H., Chen L., Wan L., Liu Q. (2018). Activity Dependent LoNA Regulates Translation by Coordinating RRNA Transcription and Methylation. Nat. Commun..

[41] Vitali P., Kiss T. (2019). Cooperative 2′-O-Methylation of the Wobble Cytidine of Human Elongator TRNA Met (CAT) by a Nucleolar and a Cajal Body-Specific Box C/D RNP. Genes. Dev..

[42] Huang C., Shi J., Guo Y., Huang W., Huang S., Ming S., Wu X., Zhang R., Ding J., Zhao W. (2017). A SnoRNA Modulates MRNA 3′ End Processing and Regulates the Expression of a Subset of MRNAs. Nucleic Acids Res..

[43] Shi J., Huang C., Huang S., Yao C. (2018). SnoRNAs Associate with MRNA 3′ Processing Complex: New Wine in Old Bottles. RNA Biol..

[44] Sharma E., Sterne-Weiler T., O’Hanlon D., Blencowe B.J. (2016). Global Mapping of Human RNA-RNA Interactions. Mol. Cell.

[45] Falaleeva M., Surface J., Shen M., de la Grange P., Stamm S. (2015). SNORD116 and SNORD115 Change Expression of Multiple Genes and Modify Each Other’s Activity. Gene.

[46] Bortolin-Cavaillé M.L., Cavaillé J. (2012). The SNORD115 (H/MBII-52) and SNORD116 (H/MBII-85) Gene Clusters at the Imprinted Prader-Willi Locus Generate Canonical Box C/D SnoRNAs. Nucleic Acids Res..

[47] Cavaillé J. (2017). Box C/D Small Nucleolar RNA Genes and the Prader-Willi Syndrome: A Complex Interplay. Wiley Interdiscip. Rev. RNA.

[48] Taft R.J., Glazov E.A., Lassmann T., Hayashizaki Y., Carninci P., Mattick J.S. (2009). Small RNAs Derived from SnoRNAs. RNA.

[49] Brameier M., Herwig A., Reinhardt R., Walter L., Gruber J. (2011). Human Box C/D SnoRNAs with MiRNA like Functions: Expanding the Range of Regulatory RNAs. Nucleic Acids Res..

[50] Burroughs A.M., Ando Y., de Hoon M.L., Tomaru Y., Suzuki H., Hayashizaki Y., Daub C.O. (2011). Deep-Sequencing of Human Argonaute-Associated Small RNAs Provides Insight into MiRNA Sorting and Reveals Argonaute Association with RNA Fragments of Diverse Origin. RNA Biol..

[51] Ono M., Yamada K., Bensaddek D., Afzal V., Biddlestone J., Ortmann B., Mudie S., Boivin V., Scott M.S., Rocha S. (2016). Enhanced SnoMEN Vectors Facilitate Establishment of GFP-HIF-1α Protein Replacement Human Cell Lines. PLoS ONE.

[52] Ono M., Yamada K., Avolio F., Scott M.S., Van Koningsbruggen S., Barton G.J., Lamond A.I. (2010). Analysis of Human Small Nucleolar RNAs (SnoRNA) and the Development of SnoRNA Modulator of Gene Expression Vectors. Mol. Biol. Cell.

[53] Yin Q.F., Yang L., Zhang Y., Xiang J.F., Wu Y.W., Carmichael G.G., Chen L.L. (2012). Long Noncoding RNAs with SnoRNA Ends. Mol. Cell.

[54] Tang Y., Chen K., Song B., Ma J., Wu X., Xu Q., Wei Z., Su J., Liu G., Rong R. (2021). M6A-Atlas: A Comprehensive Knowledgebase for Unraveling the N6-Methyladenosine (M6A) Epitranscriptome. Nucleic Acids Res..

[55] Liu H., Wang H., Wei Z., Zhang S., Hua G., Zhang S.-W., Zhang L., Gao S.-J., Meng J., Chen X. (2018). MeT-DB V2.0: Elucidating Context-Specific Functions of N6-Methyl-Adenosine Methyltranscriptome. Nucleic Acids Res..

[56] Chang G., Leu J.-S., Ma L., Xie K., Huang S. (2018). Methylation of RNA N6-Methyladenosine in Modulation of Cytokine Responses and Tumorigenesis. Cytokine.

[57] Wang X., Zhao B.S., Roundtree I.A., Lu Z., Han D., Ma H., Weng X., Chen K., Shi H., He C. (2015). N6-Methyladenosine Modulates Messenger RNA Translation Efficiency. Cell.

[58] Wang X., Lu Z., Gomez A., Hon G.C., Yue Y., Han D., Fu Y., Parisien M., Dai Q., Jia G. (2014). N6-Methyladenosine-Dependent Regulation of Messenger RNA Stability. Nature.

[59] Liu N., Dai Q., Zheng G., He C., Parisien M., Pan T. (2015). N6 -Methyladenosine-Dependent RNA Structural Switches Regulate RNA-Protein Interactions. Nature.

[60] Zhu Z., Huo F., Zhang J., Shan H., Pei D. (2023). Crosstalk between M6A Modification and Alternative Splicing during Cancer Progression. Clin. Transl. Med..

[61] Louloupi A., Ntini E., Conrad T., Ørom U.A.V. (2018). Transient N-6-Methyladenosine Transcriptome Sequencing Reveals a Regulatory Role of M6A in Splicing Efficiency. Cell Rep..

[62] Pendleton K.E., Chen B., Liu K., Hunter O.V., Xie Y., Tu B.P., Conrad N.K. (2017). The U6 SnRNA m 6 A Methyltransferase METTL16 Regulates SAM Synthetase Intron Retention. Cell.

[63] Lafaille F.G., Harschnitz O., Lee Y.S., Zhang P., Hasek M.L., Kerner G., Itan Y., Ewaleifoh O., Rapaport F., Carlile T.M. (2019). Human SNORA31 Variations Impair Cortical Neuron-Intrinsic Immunity to HSV-1 and Underlie Herpes Simplex Encephalitis. Nat. Med..

[64] Pauli C., Liu Y., Rohde C., Cui C., Fijalkowska D., Gerloff D., Walter C., Krijgsveld J., Dugas M., Edemir B. (2020). Site-Specific Methylation of 18S Ribosomal RNA by SNORD42A Is Required for Acute Myeloid Leukemia Cell Proliferation. Blood.

[65] Siprashvili Z., Webster D.E., Johnston D., Shenoy R.M., Ungewickell A.J., Bhaduri A., Flockhart R., Zarnegar B.J., Che Y., Meschi F. (2016). The Noncoding RNAs SNORD50A and SNORD50B Bind K-Ras and Are Recurrently Deleted in Human Cancer. Nat. Genet..

[66] Yoshida K., Toden S., Weng W., Shigeyasu K., Miyoshi J., Turner J., Nagasaka T., Ma Y., Takayama T., Fujiwara T. (2017). SNORA21–An Oncogenic Small Nucleolar RNA, with a Prognostic Biomarker Potential in Human Colorectal Cancer. EBioMedicine.

[67] Filippova J.A., Matveeva A.M., Zhuravlev E.S., Balakhonova E.A., Prokhorova D.V., Malanin S.J., Shah Mahmud R., Grigoryeva T.V., Anufrieva K.S., Semenov D.V. (2019). Are Small Nucleolar RNAs “CRISPRable”? A Report on Box C/D Small Nucleolar RNA Editing in Human Cells. Front. Pharmacol..

[68] Sherman B.T., Hao M., Qiu J., Jiao X., Baseler M.W., Lane H.C., Imamichi T., Chang W. (2022). DAVID: A Web Server for Functional Enrichment Analysis and Functional Annotation of Gene Lists (2021 Update). Nucleic Acids Res..

[69] Xie Z., Bailey A., Kuleshov M.V., Clarke D.J.B., Evangelista J.E., Jenkins S.L., Lachmann A., Wojciechowicz M.L., Kropiwnicki E., Jagodnik K.M. (2021). Gene Set Knowledge Discovery with Enrichr. Curr. Protoc..

[70] Chen E.Y., Tan C.M., Kou Y., Duan Q., Wang Z., Meirelles G.V., Clark N.R., Ma’ayan A. (2013). Enrichr: Interactive and Collaborative HTML5 Gene List Enrichment Analysis Tool. BMC Bioinform..

[71] Kuleshov M.V., Jones M.R., Rouillard A.D., Fernandez N.F., Duan Q., Wang Z., Koplev S., Jenkins S.L., Jagodnik K.M., Lachmann A. (2016). Enrichr: A Comprehensive Gene Set Enrichment Analysis Web Server 2016 Update. Nucleic Acids Res..

[72] Shangguan Y., Han J., Su H. (2020). GAS5 Knockdown Ameliorates Apoptosis and Inflammatory Response by Modulating MiR-26b-5p/Smad1 Axis in Cerebral Ischaemia/Reperfusion Injury. Behav. Brain Res..

[73] Zhang Y., Lu X., Yang M., Shangguan J., Yin Y. (2021). GAS5 Knockdown Suppresses Inflammation and Oxidative Stress Induced by Oxidized Low-Density Lipoprotein in Macrophages by Sponging MiR-135a. Mol. Cell Biochem..

[74] Cao L., Chen J., Ou B., Liu C., Zou Y., Chen Q. (2017). GAS5 Knockdown Reduces the Chemo-Sensitivity of Non-Small Cell Lung Cancer (NSCLC) Cell to Cisplatin (DDP) through Regulating MiR-21/PTEN Axis. Biomed. Pharmacother..

[75] Kaur J., Salehen N., Norazit A., Rahman A.A., Murad N.A.A., Rahman N.M.A.N.A., Ibrahim K. (2022). Tumor Suppressive Effects of GAS5 in Cancer Cells. Noncoding RNA.

[76] Liu Z., Wang W., Jiang J., Bao E., Xu D., Zeng Y., Tao L., Qiu J. (2013). Downregulation of GAS5 Promotes Bladder Cancer Cell Proliferation, Partly by Regulating CDK6. PLoS ONE.

[77] Shi X., Sun M., Liu H., Yao Y., Kong R., Chen F., Song Y. (2015). A Critical Role for the Long Non-coding RNA GAS5 in Proliferation and Apoptosis in Non-small-cell Lung Cancer. Mol. Carcinog..

[78] Lin G., Wu T., Gao X., He Z., Nong W. (2022). Research Progress of Long Non-Coding RNA GAS5 in Malignant Tumors. Front. Oncol..

[79] Sun D., Yu Z., Fang X., Liu M., Pu Y., Shao Q., Wang D., Zhao X., Huang A., Xiang Z. (2017). LncRNA GAS5 Inhibits Microglial M2 Polarization and Exacerbates Demyelination. EMBO Rep..

[80] Zhao D., Li Y., Yu M. (2019). LncRNA GAS5 Facilitates Nasopharyngeal Carcinoma Progression through Epigenetically Silencing PTEN via EZH2. RSC Adv..

[81] Wang M., Guo C., Wang L., Luo G., Huang C., Li Y., Liu D., Zeng F., Jiang G., Xiao X. (2018). Long Noncoding RNA GAS5 Promotes Bladder Cancer Cells Apoptosis through Inhibiting EZH2 Transcription. Cell Death Dis..

[82] Jin C., Zhao J., Zhang Z.-P., Wu M., Li J., Xiao G.-L., Liu B., Liao Y.-X., Liu J.-P. (2020). Long Non-Coding RNA GAS5, by up-Regulating PRC2 and Targeting the Promoter Methylation of MiR-424, Suppresses Multiple Malignant Phenotypes of Glioma. J. Neuro-Oncol..

[83] Kufel J., Grzechnik P. (2019). Small Nucleolar RNAs Tell a Different Tale. Trends Genet..

[84] Coker H., Wei G., Brockdorff N. (2019). M6A Modification of Non-Coding RNA and the Control of Mammalian Gene Expression. Biochim. Biophys. Acta (BBA)-Gene Regul. Mech..

[85] Parker M.T., Soanes B.K., Kusakina J., Larrieu A., Knop K., Joy N., Breidenbach F., Sherwood A.V., Barton G.J., Fica S.M. (2022). M6A Modification of U6 SnRNA Modulates Usage of Two Major Classes of Pre-MRNA 5′ Splice Site. Elife.

[86] Huang L., Ashraf S., Wang J., Lilley D.M. (2017). Control of Box C/D SnoRNP Assembly by N6-Methylation of Adenine. EMBO Rep..

[87] Ran F.A., Hsu P.D., Wright J., Agarwala V., Scott D.A., Zhang F. (2013). Genome Engineering Using the CRISPR-Cas9 System. Nat. Protoc..

[88] Brinkman E.K., Chen T., Amendola M., van Steensel B. (2014). Easy Quantitative Assessment of Genome Editing by Sequence Trace Decomposition. Nucleic Acids Res..

[89] Schroeder A., Mueller O., Stocker S., Salowsky R., Leiber M., Gassmann M., Lightfoot S., Menzel W., Granzow M., Ragg T. (2006). The RIN: An RNA Integrity Number for Assigning Integrity Values to RNA Measurements. BMC Mol. Biol..

[90] Andrews S. FastQC: A Quality Control Tool for High Throughput Sequence Data. http://www.bioinformatics.babraham.ac.uk/projects/fastqc/.

[91] Ewels P., Magnusson M., Lundin S., Käller M. (2016). MultiQC: Summarize Analysis Results for Multiple Tools and Samples in a Single Report. Bioinformatics.

[92] Chen S., Zhou Y., Chen Y., Gu J. (2018). Fastp: An Ultra-Fast All-in-One FASTQ Preprocessor. Bioinformatics.

[93] Kopylova E., Noé L., Touzet H. (2012). SortMeRNA: Fast and Accurate Filtering of Ribosomal RNAs in Metatranscriptomic Data. Bioinformatics.

[94] Dobin A., Davis C.A., Schlesinger F., Drenkow J., Zaleski C., Jha S., Batut P., Chaisson M., Gingeras T.R. (2013). STAR: Ultrafast Universal RNA-Seq Aligner. Bioinformatics.

[95] Trapnell C., Roberts A., Goff L., Pertea G., Kim D., Kelley D.R., Pimentel H., Salzberg S.L., Rinn J.L., Pachter L. (2012). Differential Gene and Transcript Expression Analysis of RNA-Seq Experiments with TopHat and Cufflinks. Nat. Protoc..

[96] Katz Y., Wang E.T., Silterra J., Schwartz S., Wong B., Thorvaldsdóttir H., Robinson J.T., Mesirov J.P., Airoldi E.M., Burge C.B. (2015). Quantitative Visualization of Alternative Exon Expression from RNA-Seq Data. Bioinformatics.

[97] Thorvaldsdottir H., Robinson J.T., Mesirov J.P. (2013). Integrative Genomics Viewer (IGV): High-Performance Genomics Data Visualization and Exploration. Brief. Bioinform..

[98] Athar A., Füllgrabe A., George N., Iqbal H., Huerta L., Ali A., Snow C., Fonseca N.A., Petryszak R., Papatheodorou I. (2019). ArrayExpress Update–From Bulk to Single-Cell Expression Data. Nucleic Acids Res..

