# Supplementary material for: Intron Editing Reveals SNORD-Dependent Maturation of the Small Nucleolar RNA Host Gene GAS5 in Human Cells
Source: Int J Mol Sci. 2023 Dec 18;24(24):17621. doi: 10.3390/ijms242417621 (PMC10743478; doi:10.3390/ijms242417621)
Supplement: Supplementary file 1 [file ijms-24-17621-s001.zip › ijms-2719824-supplementary.pdf]

Supplementary Materials

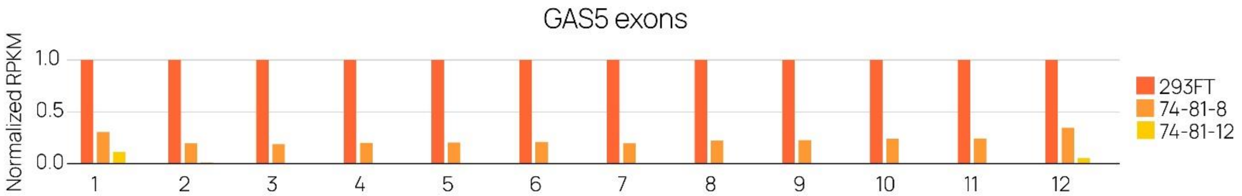

Supplementary Figure S1. The analysis of GAS5 lncRNA expression in modified cell lines exon by exon from the RNA-Seq data on polyA fraction of the transcriptome. The relative decrease in the amount of all twelve exons is present in case of both 74-81 cell lines. Data is presented as RPKMs normalized to the control 293FT cell line.

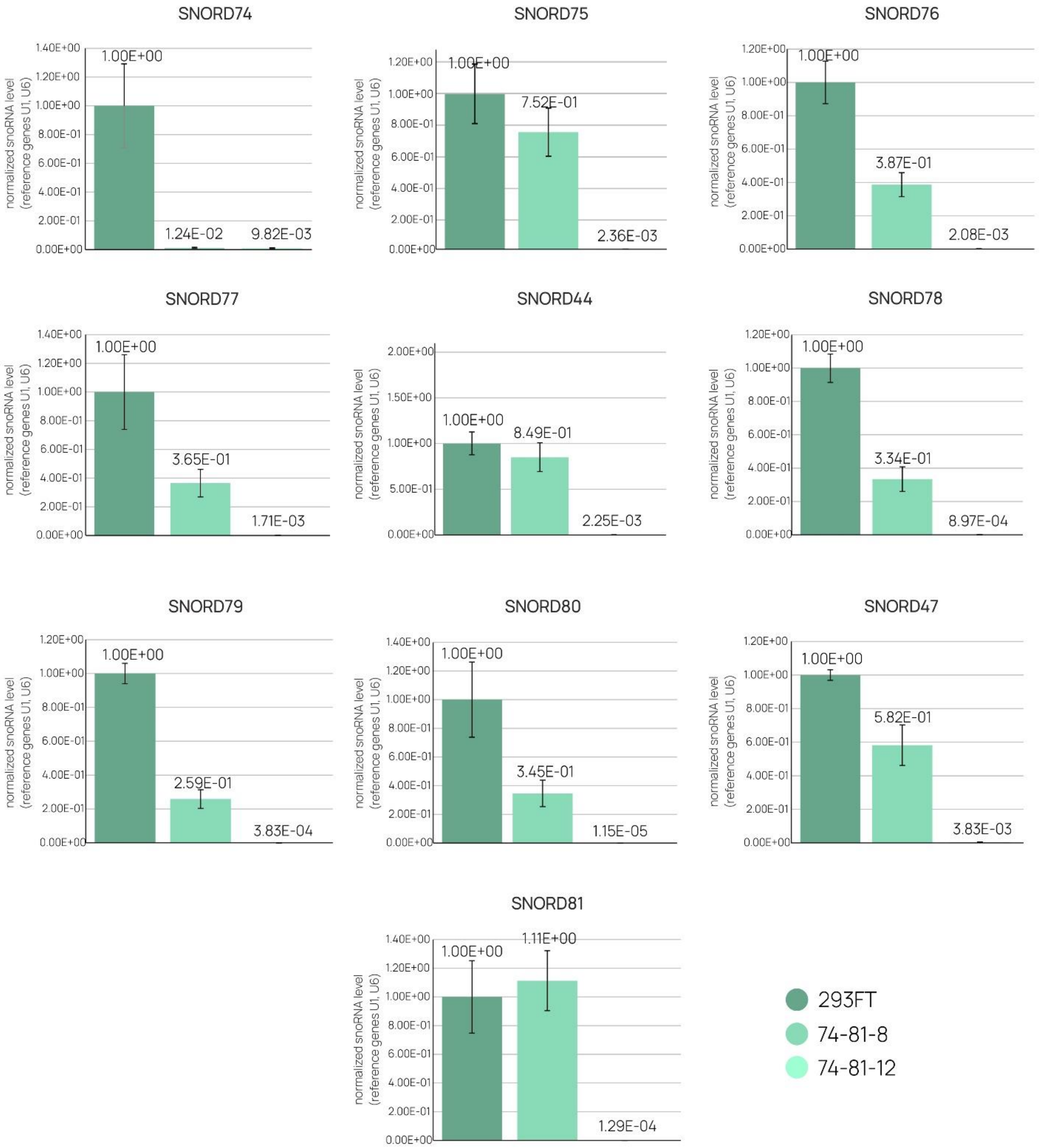

Supplementary Figure S2. Analysis of snoRNA expression in modified cell lines. 10 GAS5-encoded box-C/D-snoRNAs level was assessed using qRT-PCR with detection in real-time. U1, U6 snRNA genes were used as references. The normalized to control 293FT cell line level is presented.

Supplementary Table S1. DAVID and ENRICHR analysis and functional annotations for the upregulated DEGs. Table sections are colored in coordination with the Figure 8B.

<table>
<tr><th>Cell line</th><th>Category</th><th>Term</th><th>Genes</th><th>p-value</th></tr>
<tr><td rowspan="9">74-81-8</td><td colspan="4">DAVID Functional Annotation Chart</td></tr>
<tr><td>GOTERM_MF_DIRECT</td><td>GO:0005198~structural molecule activity</td><td>KRT17, KRT75, CRYAB</td><td>0,012</td></tr>
<tr><td>GOTERM_BP_DIRECT</td><td>GO:0031069~hair follicle morphogenesis</td><td>KRT17, FOXE1</td><td>0,026</td></tr>
<tr><td>GOTERM_CC_DIRECT</td><td>GO:0001533~cornified envelope</td><td>KRT17, KRT75</td><td>0,046</td></tr>
<tr><td>UP_SEQ_FEATURE</td><td>REGION: Coil 2</td><td>KRT17, KRT75</td><td>0,049</td></tr>
<tr><td>UP_SEQ_FEATURE</td><td>REGION: Linker 12</td><td>KRT17, KRT75</td><td>0,049</td></tr>
<tr><td>INTERPRO</td><td>IPR018039: Intermediate filament protein, conserved site</td><td>KRT17, KRT75</td><td>0,049</td></tr>
<tr><td colspan="4">ENCODE_and_ChEA_Consensus_TFs_from_ChIP-X_table</td></tr>
<tr><td colspan="2">SUZ12 CHEA</td><td>EPHA6, FOXE1, PIGZ, CD24, CACNG4</td><td>0,022</td></tr>
<tr><td rowspan="14">74-81-12</td><td colspan="4">DAVID Functional Annotation Chart</td></tr>
<tr><td>GOTERM_CC_DIRECT</td><td>GO:0009986~cell surface</td><td>SCARA5, LIPG, LPAR2, CD24, CACNG4</td><td>0,001</td></tr>
<tr><td>GOTERM_CC_DIRECT</td><td>GO:0005887~integral component of plasma membrane</td><td>ASIC4, SCARA5, LPAR2, RHBDL1, CACNG4, DRD4</td><td>0,003</td></tr>
<tr><td>UP_KW_DOMAIN</td><td>KW-1133~Transmembrane helix</td><td>RNF112, ASIC4, SCARA5, RDH16, LPAR2, MMD2, PKD1L2, RHBDL1, CACNG4, DRD4, VSTM2B</td><td>0,008</td></tr>
<tr><td>UP_KW_DOMAIN</td><td>KW-0812~Transmembrane</td><td>RNF112, ASIC4, SCARA5, RDH16, LPAR2, MMD2, PKD1L2, RHBDL1, CACNG4, DRD4, VSTM2B</td><td>0,009</td></tr>
<tr><td>UP_KW_BIOLOGICAL_PROCESS</td><td>KW-0406~Ion transport</td><td>ASIC4, SCARA5, PKD1L2, CACNG4</td><td>0,012</td></tr>
<tr><td>UP_SEQ_FEATURE</td><td>TOPO_DOM: Cytoplasmic</td><td>ASIC4, SCARA5, LPAR2, MMD2, PKD1L2, CACNG4, DRD4, VSTM2B</td><td>0,013</td></tr>
<tr><td>UP_SEQ_FEATURE</td><td>TOPO_DOM: Extracellular</td><td>ASIC4, SCARA5, LPAR2, PKD1L2, CACNG4, DRD4, VSTM2B</td><td>0,014</td></tr>
<tr><td>SMART</td><td>SM00308:LH2</td><td>LIPG, PKD1L2</td><td>0,015</td></tr>
<tr><td>UP_SEQ_FEATURE</td><td>DOMAIN: PLAT</td><td>LIPG, PKD1L2</td><td>0,016</td></tr>
<tr><td>INTERPRO</td><td>IPR001024: Lipoxygenase, LH2</td><td>LIPG, PKD1L2</td><td>0,016</td></tr>
<tr><td>GOTERM_BP_DIRECT</td><td>GO:0001662~behavioral fear response</td><td>ASIC4, DRD4</td><td>0,021</td></tr>
<tr><td>UP_SEQ_FEATURE</td><td>TRANSMEM: Helical</td><td>RNF112, ASIC4, RDH16, LPAR2, MMD2, PKD1L2, RHBDL1, CACNG4, VSTM2B</td><td>0,025</td></tr>
</table>

	UP_SEQ_FEATURE	CARBOHYD: N-linked (GlcNAc...) asparagine	ASIC4, SCARA5, LIPG, LPAR2, PKD1L2, CD24, CACNG4, DRD4	0,026
	UP_KW_CELLULAR_COMPONENT	KW-0472~Membrane	RNF112, ASIC4, SCARA5, RDH16, LPAR2, MMD2, PKD1L2, CD24, RHBDL1, CACNG4, DRD4, VSTM2B	0,033
	GOTERM_CC_DIRECT	GO:0098978~glutamatergic synapse	LPAR2, CACNG4, DRD4	0,037
	**ENCODE_and_ChEA_Consensus_TFs_from_ChIP-X_table**			
	SUZ12 CHEA		LIPG, CD24, DRD4, CACNG4, VSTM2B	0,022
	**ENCODE TF ChIP-seq 2015**			
	EZH2 B cell hg19		LIPG, YPEL4, DRD4, RHBDL1, CACNG4, VSTM2B	0,011
	EZH2 skeletal muscle myoblast hg19		SCARA5, LIPG, MMD2, CD24, CACNG4	0,043
	**DAVID Functional Annotation Chart**			
74-4-3	INTERPRO	IPR021156: Transcription elongation factor A-like/Brain expressed X-linked-like	TCEAL7, BEX5	0,012
	UP_KW_PTM	KW-0165~Cleavage on pair of basic residues	CALCB, ADAMTS18, OSTN	0,030
	UP_KW_PTM	KW-0027~Amidation	CALCB, OSTN	0,045
	UP_KW_MOLECULAR_FUNCTION	KW-0401~Integrin	ITGA4, ADAMTS18	0,047
	**ENCODE_and_ChEA_Consensus_TFs_from_ChIP-X_table**			
	SMAD4 CHEA		ITGA4, LDB2, PCDH19	0,020
74-4-4	**DAVID Functional Annotation Chart**			
	UP_SEQ_FEATURE	CARBOHYD: N-linked (GlcNAc...) asparagine	NELL1, F8, CD40, IGLON5, CCL2, GABRE, KCNK2, MXRA5, DUOX2, GBGT1, ASTN1	0,0009
	UP_KW_PTM	KW-0325~Glycoprotein	NELL1, F8, CD40, CTNND2, IGLON5, CCL2, GABRE, KCNK2, MXRA5, DUOX2, GBGT1, ASTN1	0,0020
	UP_KW_PTM	KW-1015~Disulfide bond	NELL1, F8, CD40, IGLON5, CCL2, GABRE, KCNK2, MXRA5, DUOX2, ASTN1	0,0076
	UP_KW_DOMAIN	KW-0732~Signal	NELL1, F8, CD40, IGLON5, CCL2, SOX11, GABRE, MXRA5, DUOX2, ASTN1	0,0089
	GOTERM_BP_DIRECT	GO:0045778~positive regulation of ossification	NELL1, SOX11	0,0122
	GOTERM_MF_DIRECT	GO:0016491~oxidoreductase activity	F8, NOS1, VAT1L	0,0124
	UP_SEQ_FEATURE	DOMAIN: FAD-binding FR-type	NOS1, DUOX2	0,0172

	INTERPRO	IPR017927: Ferredoxin reductase-type FAD-binding domain	NOS1, DUOX2	0,0174
	INTERPRO	IPR017938: Riboflavin synthase-like beta-barrel	NOS1, DUOX2	0,0183
	GOTERM_BP_DIRECT	GO:2000353~positive regulation of endothelial cell apoptotic process	CD40, CCL2	0,0185
	UP_KW_DOMAIN	KW-0677~Repeat	SPRR2F, NELL1, F8, CD40, CTNND2, IGLON5, KLHL4, MXRA5, DUOX2, ASTN1	0,0224
	BBID	19.Cytokine_microglia	CD40, CCL2	0,0284
	UP_KW_CELLULAR_COMPONENT	KW-0964~Secreted	NELL1, F8, CD40, IGLON5, CCL2, MXRA5	0,0360
	GOTERM_BP_DIRECT	GO:0007399~nervous system development	NELL1, BEX1, SOX11	0,0364
	KEGG_PATHWAY	hsa05144: Malaria	CD40, CCL2	0,0457
	**ENCODE_and_ChEA_Consensus_TFs_from_ChIP-X_table**			
	SUZ12 CHEA		NELL1, CTNND2, SOX11, KCNK2, DUOX2, VAT1L	0,005
	REST CHEA		NELL1, CTNND2, NOS1, VAT1L, ASTN1	0,007
	EGR1 CHEA		NOS1, KCNK2	0,039
	TP63 CHEA		CD40, CTNND2, NOS1, MXRA5	0,047
81-6	**DAVID Functional Annotation Chart**			
	GOTERM_CC_DIRECT	GO:0009986~cell surface	SRPX2, SCARA5, CD24, CRYAB, CD44	0,001
	GOTERM_CC_DIRECT	GO:0070062~extracellular exosome	GPRC5A, PCK1, KRT75, CRYAB, ALDH1L2, CD44, RHOB	0,005
	GOTERM_BP_DIRECT	GO:0098609~cell-cell adhesion	SRPX2, CD24, CD44	0,011
	GOTERM_BP_DIRECT	GO:0042325~regulation of phosphorylation	SRPX2, CD24	0,013
	GOTERM_BP_DIRECT	GO:0001525~angiogenesis	SRPX2, ADM2, RHOB	0,018
	GOTERM_BP_DIRECT	GO:0016477~cell migration	CD24, CD44, RHOB	0,021
	GOTERM_CC_DIRECT	GO:0097060~synaptic membrane	SRPX2, CRYAB	0,031
	GOTERM_BP_DIRECT	GO:0043154~negative regulation of cysteine-type endopeptidase	CRYAB, CD44	0,045

		activity involved in apoptotic process		
	UP_KW_CELLULAR_COMPONENT	KW-1003~Cell membrane	GPRC5A, SCARA5, SH2D3C, SLCO5A1, CD24, PDE9A, CD44, RHOB	0,047
	**ENCODE_and_ChEA_Consensus_TFs_from_ChIP-X_table**			
	SOX2 CHEA		SLCO5A1, CD24, KLF2, RHOB	0,007
	**ENCODE TF ChIP-seq 2015**			
	EZH2 skeletal muscle myoblast hg19		SCARA5, ADM2, SLCO5A1, C6ORF141, CD24, CRYAB, PDE9A	0,002
	MEF2C GM12878 hg19		KLF2, RHOB	0,010
	FOS endothelial cell of umbilical vein hg19		C11ORF96, SRPX2, GPRC5A, CD44, RHOB	0,043
	CBX8 K562 hg19		C11ORF96, SRPX2, GPRC5A, ALDH1L2, PDE9A	0,043

Supplementary Table S2. DAVID and ENRICHR analysis and functional annotations for the downregulated DEGs. Table sections are colored in coordination with the Figure 8C.

Cell line	Category	Term	Genes	p-value
	**DAVID Functional Annotation Chart**			
	UP_KW_PTM	KW-1015~Disulfide bond	TENM1, RAMP2, ENPEP, NRROS, CFI, FZD10, SLC7A11, ADAMTS12, EDNRA, SPOCK3, SOSTDC1, OXGR1, GABRQ, SRGN, LUM, VEGFC, GPR50, INHBE, POMC, GALC, XK, SLCO3A1, FABP5, SCO2, PXDN, MXRA8, FOSB, LGR5, TLR3, GPR19	7,93E-05
	UP_KW_PTM	KW-0325~Glycoprotein	TENM1, RAMP2, ENPEP, NRROS, PCDH10, CFI, FZD10, SLC7A11, ADAMTS12, EDNRA, GPNMB, SPOCK3, SOSTDC1, QRFPR, OXGR1, GABRQ, SRGN, SLC10A4, LUM, P3H2, VEGFC, INHBE, POMC, GALC, SLCO3A1, DCT, SELENOP, PXDN, MXRA8, COL21A1, LGR5, TLR3, TRABD2A, GPR19	9,12E-05
	UP_SEQ_FEATURE	REPEAT: LRR 11	NRROS, LUM, LGR5, FBXL7, TLR3	0,0005
	UP_SEQ_FEATURE	REPEAT: LRR 8	NRROS, LUM, PXDN, LGR5, FBXL7, TLR3	0,0007
	UP_SEQ_FEATURE	DOMAIN: LRRNT	NRROS, LUM, PXDN, LGR5, TLR3	0,0008
74-81-8	UP_SEQ_FEATURE	REPEAT: LRR 10	NRROS, LUM, LGR5, FBXL7, TLR3	0,0010
	UP_SEQ_FEATURE	REPEAT: LRR 7	NRROS, LUM, PXDN, LGR5, FBXL7, TLR3	0,0015
	UP_SEQ_FEATURE	CARBOHYD: N-linked (GlcNAc...) asparagine	TENM1, RAMP2, ENPEP, NRROS, PCDH10, CFI, FZD10, SLC7A11, ADAMTS12, EDNRA, GPNMB, SOSTDC1, QRFPR, OXGR1, GABRQ, SLC10A4, P3H2, VEGFC, INHBE, POMC, GALC, SLCO3A1, DCT, SELENOP, PXDN, MXRA8, COL21A1, LGR5, TLR3, TRABD2A, GPR19	0,0015
	GOTERM_CC_DIRECT	GO:0005887~integral component of plasma membrane	GABRQ, TENM1, ENPEP, RAMP2, PCDH10, GPR50, FZD10, EDNRA, SLCO3A1, GPNMB, QRFPR, LGR5, TLR3, TRABD2A, GPR19	0,0019
	UP_SEQ_FEATURE	REPEAT: LRR 6	NRROS, LUM, PXDN, LGR5, FBXL7, TLR3	0,0024
	GOTERM_MF_DIRECT	GO:0005324~long-chain fatty acid transporter activity	FABP5, SLC27A6, SLC27A5	0,0025
	GOTERM_BP_DIRECT	GO:0030199~collagen fibril organization	LUM, PXDN, COL21A1, ADAMTS12	0,0038

	UP_SEQ_FEATURE	REPEAT: LRR 9	NRROS, LUM, LGR5, FBXL7, TLR3	0,0039
	UP_SEQ_FEATURE	REPEAT: LRR 5	NRROS, LUM, PXDN, LGR5, FBXL7, TLR3	0,0042
	UP_SEQ_FEATURE	REPEAT: LRR 4	NRROS, LUM, PXDN, LGR5, FBXL7, TLR3	0,0043
	GOTERM_BP_DIRECT	GO:0008217~regulation of blood pressure	POMC, EDNRA, RAMP2, ENPEP	0,0047
	SMART	SM00369: LRR_TYP	NRROS, LUM, PXDN, LGR5, TLR3	0,0049
	UP_SEQ_FEATURE	REPEAT: LRR 3	NRROS, LUM, PXDN, LGR5, FBXL7, TLR3	0,0053
	INTERPRO	IPR001611: Leucine-rich repeat	NRROS, LUM, PXDN, LGR5, FBXL7, TLR3	0,0057
	UP_SEQ_FEATURE	REPEAT: LRR 2	NRROS, LUM, PXDN, LGR5, FBXL7, TLR3	0,0060
	UP_KW_DOMAIN	KW-0732~Signal	RAMP2, NRROS, PCDH10, CFI, FZD10, ADAMTS12, EDNRA, GPNMB, SPOCK3, SOSTDC1, LRAT, GABRQ, IL32, SRGN, LUM, P3H2, VEGFC, INHBE, POMC, GALC, XK, SLCO3A1, DCT, SELENOP, PXDN, MXRA8, COL21A1, LGR5, TLR3, TRABD2A	0,0061
	UP_SEQ_FEATURE	REPEAT: LRR 1	NRROS, LUM, PXDN, LGR5, FBXL7, TLR3	0,0063
	GOTERM_BP_DIRECT	GO:0007165~signal transduction	GABRQ, IL32, TENM1, ENPEP, VEGFC, RERG, ARHGAP24, INHBE, POMC, EDNRA, GPNMB, PDE3A, TLR3	0,0063
	UP_KW_PTM	KW-0165~Cleavage on pair of basic residues	POMC, TENM1, CFI, VEGFC, ADAMTS12, INHBE	0,0067
	INTERPRO	IPR003591: Leucine-rich repeat, typical subtype	NRROS, LUM, PXDN, LGR5, TLR3	0,0075
	GOTERM_BP_DIRECT	GO:0034346~positive regulation of type III interferon production	IL32, TLR3	0,0081
	GOTERM_BP_DIRECT	GO:1990830~cellular response to leukemia inhibitory factor	MAT2A, VEGFC, LRAT, TEX14	0,0094
	UP_SEQ_FEATURE	REPEAT: LRR 16	NRROS, LGR5, TLR3	0,0108
	UP_KW_DOMAIN	KW-0433~Leucine-rich repeat	NRROS, LUM, PXDN, LGR5, FBXL7, TLR3	0,0118
	GOTERM_BP_DIRECT	GO:0032870~cellular response to hormone stimulus	RAMP2, FOSB, QRFPR	0,0132
	UP_SEQ_FEATURE	REPEAT: LRR 15	NRROS, LGR5, TLR3	0,0154
	UP_SEQ_FEATURE	REPEAT: LRR 14	NRROS, LGR5, TLR3	0,0161

	UP_SEQ_FEATURE	DOMAIN: Kazal-like	SLCO3A1, CFI, SPOCK3	0,0180
	INTERPRO	IPR002350: Kazal domain	SLCO3A1, CFI, SPOCK3	0,0202
	GOTERM_CC_DIRECT	GO:0031012~extracellular matrix	LUM, SPOCK3, PXDN, ADAMTS12, TLR3	0,0205
	INTERPRO	IPR013024: Butirosin biosynthesis, BtrG-like	CHAC1, GGACT	0,0213
	UP_SEQ_FEATURE	TRANSMEM: Helical	TENM1, RAMP2, ENPEP, NRROS, PCDH10, FZD10, SLC7A11, NKAIN2, GPNMB, MYADML2, LRAT, QRFPR, OXGR1, GABRQ, SLC10A4, GPR50, CYP7B1, XK, SLCO3A1, DCT, ACTBL2, FES, SCO2, SELENOP, PDE3A, MXRA8, SLC27A6, LGR5, SLC27A5, TLR3, TRABD2A, GPR19	0,0213
	UP_SEQ_FEATURE	REPEAT:LRR 13	NRROS, LGR5, TLR3	0,0222
	UP_SEQ_FEATURE	TOPO_DOM: Extracellular	GABRQ, SLC10A4, TENM1, ENPEP, RAMP2, NRROS, PCDH10, GPR50, SLC7A11, FZD10, EDNRA, XK, SLCO3A1, GPNMB, MXRA8, QRFPR, LGR5, TRABD2A, GPR19, OXGR1	0,0260
	GOTERM_CC_DIRECT	GO:0005615~extracellular space	SRGN, IL32, LUM, CFI, VEGFC, SERPINB8, INHBE, POMC, ACTBL2, FABP5, SPOCK3, PXDN, SOSTDC1, COL21A1, TLR3	0,0265
	GOTERM_BP_DIRECT	GO:0007155~cell adhesion	IL32, GPNMB, FES, PCDH10, PXDN, MXRA8, SLC7A11	0,0276
	GOTERM_MF_DIRECT	GO:0015245~fatty acid transporter activity	SLC27A6, SLC27A5	0,0306
	GOTERM_BP_DIRECT	GO:0048066~developmental pigmentation	EDNRA, DCT	0,0320
	UP_KW_CELLULAR_COMPONENT	KW-0272~Extracellular matrix	LUM, SPOCK3, PXDN, COL21A1, ADAMTS12	0,0323
	UP_SEQ_FEATURE	REPEAT: LRR 12	NRROS, LGR5, TLR3	0,0378
	GOTERM_BP_DIRECT	GO:0097084~vascular smooth muscle cell development	EDNRA, RAMP2	0,0399
	GOTERM_BP_DIRECT	GO:0007186~G-protein coupled receptor signaling pathway	EDNRA, RAMP2, PDE3A, GPR50, FZD10, QRFPR, LGR5, GPR19, OXGR1	0,0423
	GOTERM_MF_DIRECT	GO:0031957~very long-chain fatty	SLC27A6, SLC27A5	0,0434

		acid-CoA ligase activity		
	UP_SEQ_FEATURE	REPEAT: LRR 21	NRROS, TLR3	0,0473
	GOTERM_BP_DIRECT	GO:0070831~basement membrane assembly	RAMP2, PXDN	0,0477
	**ENCODE_and_ChEA_Consensus_TFs_from_ChIP-X_table**			
	CEBPB ENCODE		SLC7A11, CHAC1	0,009
	SUZ12 CHEA		EGR3, SPOCK3, QRFPR, RERG, FBXL7	0,022
	**WikiPathway_2023_Human_table**			
	P53 Transcriptional Gene Network WP4963		SCO2, SLC7A11, MLH1	9,82E-05
	mRNA Protein And Metabolite Inducation Pathway By Cyclosporin A WP3953		SLC7A11	0,007
	Amino Acid Metabolism In Triple Negative Breast Cancer Cells WP5213		SLC7A11	0,007
	Transcriptional Activation By NRF2 In Response To Phytochemicals WP3		SLC7A11	0,015
	DNA Mismatch Repair WP531		MLH1	0,023
	NRF2 ARE Regulation WP4357		SLC7A11	0,023
	Antiviral And Anti Inflammatory Effects Of Nrf2 On SARS CoV 2 Pathway WP5113		SLC7A11	0,031
	Ovarian Infertility WP34		MLH1	0,036
	Nuclear Receptors Meta Pathway WP2882		SRGN;SLC7A11	0,039
	Exercise Induced Circadian Regulation WP410		G0S2	0,047
74-81-12	**DAVID Functional Annotation Chart**			
	GOTERM_BP_DIRECT	GO:0035914~skeletal muscle cell differentiation	EGR1, HLF, FOS	0,0008
	INTERPRO	IPR021849: Protein of unknown function DUF3446	EGR1, EGR3	0,0022
	GOTERM_MF_DIRECT	GO:0001228~transcriptional activator activity, RNA polymerase II transcription regulatory region sequence-specific binding	EGR1, EGR3, HLF, FOS	0,0059
	UP_SEQ_FEATURE	COMPBIAS: Basic and acidic residues	EGR1, EGR3, HLF, CCDC116, PCDH10, TCEANC, AGAP11, PDE3A, ZNF804A, NEFM, FOS	0,0067
	GOTERM_MF_DIRECT	GO:1990837~sequence-specific double-	EGR1, EGR3, HLF, FOS	0,0089

		stranded DNA binding		
	GOTERM_MF_DIRECT	GO:0003700~transcription factor activity, sequence-specific DNA binding	EGR1, EGR3, HLF, FOS	0,0117
	GOTERM_MF_DIRECT	GO:0043565~sequence-specific DNA binding	EGR1, HLF, FOS	0,0298
	GOTERM_BP_DIRECT	GO:1901216~positive regulation of neuron death	EGR1, FOS	0,0318
	UP_SEQ_FEATURE	DOMAIN: BZIP	HLF, FOS	0,0381
	INTERPRO	IPR004827: Basic-leucine zipper domain	HLF, FOS	0,0402
	GOTERM_BP_DIRECT	GO:0014823~response to activity	SCO2, FOS	0,0428
	UP_SEQ_FEATURE	DOMAIN: bZIP	HLF, FOS	0,0445
	SMART	SM00338: BRLZ	HLF, FOS	0,0451
	GOTERM_BP_DIRECT	GO:1902895~positive regulation of pri-miRNA transcription from RNA polymerase II promoter	EGR1, FOS	0,0453
	**ENCODE_and_ChEA_Consensus_TFs_from_ChIP-X_table**			
	NELFE ENCODE		EGR1, FOS, GAS5	0,0016
	ZMIZ1 ENCODE		EGR1, SCO2, TCEANC, HSF2BP, CHAC1	0,0017
	SRF ENCODE		EGR1, EGR3, FOS	0,0031
	TAF7 ENCODE		EGR1, HSF2BP, FOS, GAS5	0,0033
	MAX ENCODE		HLF, SCO2, TCEANC, HSF2BP, C6ORF52, GAS5	0,0133
	TRIM28 CHEA		EGR1, NEFM	0,0184
	CREB1 ENCODE		EGR1, EGR3, HSF2BP, FOS, C6ORF52, GAS5	0,0189
	SUZ12 CHEA		EGR3, HLF, PCDH10, PDE3A, NEFM	0,0224
	NFIC ENCODE		EGR1, FOS	0,0312
	STAT3 ENCODE		EGR1, TCEANC, FOS	0,0342
	USF1 ENCODE		SCO2, TCEANC, C6ORF52, GAS5	0,0495
	NELFE ENCODE		EGR1, FOS, GAS5	0,0016
	**BioPlanet_2019_table**			
	Regulation of NFAT transcription factors		EGR1, EGR3, FOS	1,35E-05
	Differentiation pathway in PC12 cells		EGR1, EGR3, FOS	7,74E-05
	Interleukin-5 regulation of apoptosis		EGR1, EGR3, FOS	0,0004

	MAPK/TRK pathway		EGR1, FOS	0,0005
	CD8/T cell receptor downstream pathway		EGR1, FOS	0,0021
	BDNF signaling pathway		EGR1, EGR3, FOS	0,0021
	AP-1 transcription factor network		EGR1, FOS	0,0022
	Insulin signaling pathway		EGR1, PDE3A, FOS	0,0025
	Regular glucocorticoid receptor pathway		EGR1, FOS	0,0030
74-4-3	**DAVID Functional Annotation Chart**			
	GOTERM_MF_DIRECT	GO:0052593~tryptamine: oxygen oxidoreductase (deaminating) activity	AOC3, AOC2	0,0015
	GOTERM_MF_DIRECT	GO:0052594~aminoacetone: oxygen oxidoreductase(deaminating) activity	AOC3, AOC2	0,0015
	UP_KW_CELLULAR_COMPONENT	KW-0472~Membrane	AOC3, MMP14, ANKLE1, KCND1, AOC2, SLC43A3, CTSK, GUCA1B, CDHR5, GP1BA, DRD4, CEACAM19	0,0015
	UP_SEQ_FEATURE	ACT_SITE: Schiff-base intermediate with substrate; via topaquinone	AOC3, AOC2	0,0019
	UP_SEQ_FEATURE	CARBOHYD: N-linked (GlcNAc...) asparagine	AOC3, KCND1, AOC2, SLC43A3, CTSK, CDHR5, GP1BA, DRD4, CEACAM19	0,0020
	INTERPRO	IPR015800: Copper amine oxidase, N2-terminal	AOC3, AOC2	0,0020
	INTERPRO	IPR016182: Copper amine oxidase, N-terminal	AOC3, AOC2	0,0020
	INTERPRO	IPR000269: Copper amine oxidase	AOC3, AOC2	0,0020
	INTERPRO	IPR015798: Copper amine oxidase, C-terminal	AOC3, AOC2	0,0020
	INTERPRO	IPR015802: Copper amine oxidase, N3-terminal	AOC3, AOC2	0,0020
	GOTERM_MF_DIRECT	GO:0052596~phenethylamine: oxygen oxidoreductase (deaminating) activity	AOC3, AOC2	0,0029
	GOTERM_MF_DIRECT	GO:0052595~aliphatic-amine oxidase activity	AOC3, AOC2	0,0029

	GOTERM_MF_DIRECT	GO:0008131~primary amine oxidase activity	AOC3, AOC2	0,0044
	GOTERM_BP_DIRECT	GO:0009308~amine metabolic process	AOC3, AOC2	0,0060
	UP_KW_PTM	KW-0801~TPQ	AOC3, AOC2	0,0062
	UP_KW_PTM	KW-0325~Glycoprotein	AOC3, KCND1, AOC2, SLC43A3, CTSK, CDHR5, GP1BA, DRD4, CEACAM19	0,0094
	UP_KW_LIGAND	KW-0106~Calcium	AOC3, MMP14, AOC2, GUCA1B, CDHR5	0,0096
	GOTERM_MF_DIRECT	GO:0048038~quinone binding	AOC3, AOC2	0,0103
	KEGG_PATHWAY	hsa00360: Phenylalanine metabolism	AOC3, AOC2	0,0130
	GOTERM_CC_DIRECT	GO:0016021~integral component of membrane	AOC3, MMP14, ANKLE1, KCND1, SLC43A3, CTSK, CDHR5, GP1BA, CEACAM19	0,0150
	UP_KW_DOMAIN	KW-1133~Transmembrane helix	AOC3, MMP14, ANKLE1, KCND1, SLC43A3, CTSK, CDHR5, GP1BA, DRD4, CEACAM19	0,0164
	UP_KW_DOMAIN	KW-0812~Transmembrane	AOC3, MMP14, ANKLE1, KCND1, SLC43A3, CTSK, CDHR5, GP1BA, DRD4, CEACAM19	0,0175
	UP_SEQ_FEATURE	TOPO_DOM:Cytoplasmic	AOC3, MMP14, KCND1, CDHR5, GP1BA, DRD4, CEACAM19	0,0228
	KEGG_PATHWAY	hsa00410: beta-Alanine metabolism	AOC3, AOC2	0,0250
	GOTERM_BP_DIRECT	GO:0030574~collagen catabolic process	MMP14, CTSK	0,0271
	KEGG_PATHWAY	hsa00350: Tyrosine metabolism	AOC3, AOC2	0,0290
	UP_SEQ_FEATURE	TOPO_DOM: Extracellular	AOC3, MMP14, CDHR5, GP1BA, DRD4, CEACAM19	0,0295
	GOTERM_BP_DIRECT	GO:0022617~extracellular matrix disassembly	MMP14, CTSK	0,0303
	KEGG_PATHWAY	hsa00260: Glycine, serine and threonine metabolism	AOC3, AOC2	0,0322
	UP_KW_CELLULAR_COMPONENT	KW-1003~Cell membrane	AOC3, AOC2, SLC43A3, CTSK, GUCA1B, CDHR5, DRD4	0,0329
	GOTERM_BP_DIRECT	GO:0014070~response to organic cyclic compound	MMP14, CTSK	0,0432
	GOTERM_MF_DIRECT	GO:0005507~copper ion binding	AOC3, AOC2	0,0434

	UP_SEQ_FEATURE	PROPEP: Activation peptide	MMP14, CTSK	0,0476
	**ENCODE_and_ChEA_Consensus_TFs_from_ChIP-X_table**			
	GATA1 CHEA		KCND1, SLC43A3, GP1BA	0,045
	SALL4 CHEA		SLC43A3, CTGF	0,048
74-4-4	GOTERM_BP_DIRECT	GO:0071300~cellular response to retinoic acid	YES1, BRINP3, EPHA3	0,0016
	INTERPRO	IPR020635: Tyrosine-protein kinase, catalytic domain	YES1, ERBB4, EPHA3	0,0019
	UP_SEQ_FEATURE	MOTIF: PDZ-binding	ERBB4, DLGAP1, EPHA3	0,0022
	INTERPRO	IPR008266: Tyrosine-protein kinase, active site	YES1, ERBB4, EPHA3	0,0023
	SMART	SM00219: TyrKc	YES1, ERBB4, EPHA3	0,0032
	UP_KW_MOLECULAR_FUNCTION	KW-0829~Tyrosine-protein kinase	YES1, ERBB4, EPHA3	0,0033
	INTERPRO	IPR009030: Insulin-like growth factor binding protein, N-terminal	ERBB4, BRINP3, EPHA3	0,0050
	INTERPRO	IPR001245: Serine-threonine/tyrosine-protein kinase catalytic domain	YES1, ERBB4, EPHA3	0,0051
	GOTERM_BP_DIRECT	GO:0007169~transmembrane receptor protein tyrosine kinase signaling pathway	YES1, ERBB4, EPHA3	0,0055
	UP_KW_DOMAIN	KW-0677~Repeat	ZNF595, TENM1, ZNF91, CSRP1, ERBB4, ESRP2, KRTAP19-1, ZNF334, EPHA3	0,0113
	UP_SEQ_FEATURE	ZN_FING: C2H2-type 13	ZNF595, ZNF91, ZNF334	0,0114
	UP_SEQ_FEATURE	ZN_FING: C2H2-type 12	ZNF595, ZNF91, ZNF334	0,0175
	UP_SEQ_FEATURE	ZN_FING: C2H2-type 11	ZNF595, ZNF91, ZNF334	0,0219
	UP_KW_PTM	KW-0597~Phosphoprotein	TENM1, YES1, GNAL, CSRP1, ERBB4, ESRP2, DLGAP1, CRYAB, CEP76, EPHA3, DDN	0,0264
	UP_SEQ_FEATURE	ZN_FING: C2H2-type 10	ZNF595, ZNF91, ZNF334	0,0293
	INTERPRO	IPR017441: Protein kinase, ATP binding site	YES1, ERBB4, EPHA3	0,0321
	UP_SEQ_FEATURE	ZN_FING: C2H2-type 9	ZNF595, ZNF91, ZNF334	0,0355

	UP_SEQ_FEATURE	DOMAIN: KRAB	ZNF595, ZNF91, ZNF334	0,0374
	INTERPRO	IPR001909: Krueppel-associated box	ZNF595, ZNF91, ZNF334	0,0394
	UP_KW_BIOLOGICAL_PROCESS	KW-0805~Transcription regulation	ZNF595, TENM1, ZNF91, ERBB4, ZNF334	0,0403
	GOTERM_BP_DIRECT	GO:0048013~ephrin receptor signaling pathway	YES1, EPHA3	0,0403
	UP_SEQ_FEATURE	ZN_FING: C2H2-type 8	ZNF595, ZNF91, ZNF334	0,0423
	UP_KW_BIOLOGICAL_PROCESS	KW-0804~Transcription	ZNF595, TENM1, ZNF91, ERBB4, ZNF334	0,0445
	GOTERM_MF_DIRECT	GO:0004714~transmembrane receptor protein tyrosine kinase activity	ERBB4, EPHA3	0,0446
	GOTERM_BP_DIRECT	GO:0018108~peptidyl-tyrosine phosphorylation	ERBB4, EPHA3	0,0466
	GOTERM_BP_DIRECT	GO:0007399~nervous system development	TENM1, ERBB4, BRINP3	0,0467
	UP_SEQ_FEATURE	ZN_FING: C2H2-type 7	ZNF595, ZNF91, ZNF334	0,0480
	**ENCODE TF ChIP-seq 2015**			
	NR2F2 MCF-7 hg19		ZNF595, CSRP1, ESRP2, GAS5	0,003
	EZH2 B cell hg19		ZNF595, YES1, GNAL, ESRP2, ERBB4, ZNF334	0,011
	MAFK GM12878 hg19		ZNF595, GNAL	0,016
	EZH2 mammary epithelial cell hg19		GNAL, GAS5, ZNF334, EPHA3, DDN	0,043
	POLR2A cerebellum mm9		GNAL, CSRP1, ERBB4, CRYAB, EPHA3	0,043
81-6	GOTERM_BP_DIRECT	GO:0071276~cellular response to cadmium ion	MT1F, MT1X, FOS	0,0006
	INTERPRO	IPR021849: Protein of unknown function DUF3446	EGR1, EGR3	0,0031
	INTERPRO	IPR000837: Fos transforming protein	FOSB, FOS	0,0094
	INTERPRO	IPR018064: Metallothionein, vertebrate, metal binding site	MT1F, MT1X	0,0104
	UP_SEQ_FEATURE	REGION: Beta	MT1F, MT1X	0,0122
	UP_SEQ_FEATURE	REGION: Alpha	MT1F, MT1X	0,0122

	KEGG_PATHWAY	hsa04928: Parathyroid hormone synthesis, secretion and action	EGR1, PDE4B, FOS	0,0125
	GOTERM_BP_DIRECT	GO:0071248~cellular response to metal ion	MT1F, MT1X	0,0126
	INTERPRO	IPR000006: Metallothionein, vertebrate	MT1F, MT1X	0,0135
	INTERPRO	IPR017854: Metallothionein domain	MT1F, MT1X	0,0145
	INTERPRO	IPR003019: Metallothionein superfamily, eukaryotic	MT1F, MT1X	0,0145
	INTERPRO	IPR023587: Metallothionein domain, vertebrate	MT1F, MT1X	0,0145
	GOTERM_MF_DIRECT	GO:0008270~zinc ion binding	EGR1, ENPEP, APOBEC3G, MT1F, MT1X	0,0151
	UP_SEQ_FEATURE	SITE: Interaction with DNA	EGR1, APOBEC3G	0,0152
	GOTERM_MF_DIRECT	GO:0001228~transcriptional activator activity, RNA polymerase II transcription regulatory region sequence-specific binding	EGR1, EGR3, FOSB, FOS	0,0155
	GOTERM_BP_DIRECT	GO:0015909~long-chain fatty acid transport	SLC27A6, PLIN2	0,0155
	GOTERM_BP_DIRECT	GO:0010273~detoxification of copper ion	MT1F, MT1X	0,0155
	UP_KW_LIGAND	KW-0104~Cadmium	MT1F, MT1X	0,0158
	GOTERM_BP_DIRECT	GO:0051412~response to corticosterone	FOSB, FOS	0,0174
	GOTERM_BP_DIRECT	GO:0045926~negative regulation of growth	MT1F, MT1X	0,0174
	UP_KW_LIGAND	KW-0480~Metal-thiolate cluster	MT1F, MT1X	0,0205
	GOTERM_MF_DIRECT	GO:1990837~sequence-specific double-stranded DNA binding	EGR1, EGR3, FOSB, FOS	0,0229

	GOTERM_BP_DIRECT	GO:0071294~cellular response to zinc ion	MT1F, MT1X	0,0232
	UP_SEQ_FEATURE	ACT_SITE: Proton donor	TKTL1, APOBEC3G, PDE4B	0,0250
	GOTERM_BP_DIRECT	GO:0071280~cellular response to copper ion	MT1F, MT1X	0,0260
	GOTERM_BP_DIRECT	GO:0009410~response to xenobiotic stimulus	FOSB, PLIN2, FOS	0,0294
	GOTERM_MF_DIRECT	GO:0003700~transcription factor activity, sequence-specific DNA binding	EGR1, EGR3, FOSB, FOS	0,0295
	GOTERM_BP_DIRECT	GO:0006882~cellular zinc ion homeostasis	MT1F, MT1X	0,0346
	GOTERM_BP_DIRECT	GO:0032570~response to progesterone	FOSB, FOS	0,0346
	GOTERM_BP_DIRECT	GO:1901216~positive regulation of neuron death	EGR1, FOS	0,0355
	GOTERM_BP_DIRECT	GO:0051591~response to cAMP	FOSB, FOS	0,0393
	GOTERM_BP_DIRECT	GO:0032870~cellular response to hormone stimulus	FOSB, FOS	0,0412
	GOTERM_CC_DIRECT	GO:0005634~nucleus	TKTL1, EGR1, EGR3, APOBEC3G, PDE4B, MT1F, FOSB, MT1X, PLIN2, FOS, DHRS2	0,0435
	GOTERM_BP_DIRECT	GO:0035914~skeletal muscle cell differentiation	EGR1, FOS	0,0459
	UP_SEQ_FEATURE	DOMAIN: BZIP	FOSB, FOS	0,0469
	**ENCODE_and_ChEA_Consensus_TFs_from_ChIP-X_table**			
	SRF ENCODE		EGR1, EGR3, FOSB	0,003
	**BioPlanet_2019_table**			
	BDNF signaling pathway		EGR1, EGR3, PDE4B, FOSB	0,0001
	Interleukin-5 regulation of apoptosis		EGR1, EGR3, PDE4B	0,0004
	Regulation of NFAT transcription factors		EGR1, EGR3	0,0010
	AP-1 transcription factor network		EGR1, FOSB	0,0022
	Differentiation pathway in PC12 cells		EGR1, EGR3	0,0032
